# Ultrathin, Dynamically Controllable Circularly Polarized Emission Laser Enabled by Resonant Chiral Metasurfaces

**DOI:** 10.1021/acsphotonics.4c01005

**Published:** 2024-11-22

**Authors:** Ioannis Katsantonis, Anna C. Tasolamprou, Eleftherios N. Economou, Thomas Koschny, Maria Kafesaki

**Affiliations:** † Foundation of Research and Technology Hellas, Institute of Electronic Structure and Laser, Heraklion 71110, Greece; ‡ Department of Physics, National and Kapodistrian University of Athens, Athens 15784, Greece; § Department of Physics, University of Crete, Heraklion 71003, Greece; ∥ Ames National Laboratory and Department of Physics and Astronomy, Iowa State University, Ames, Iowa 50011, United States; ⊥ Department of Material Science and Engineering University of Crete, Heraklion 71003, Greece

**Keywords:** circularly polarized laser emission, chiral-light-matter interaction, active chiral metasurfaces

## Abstract

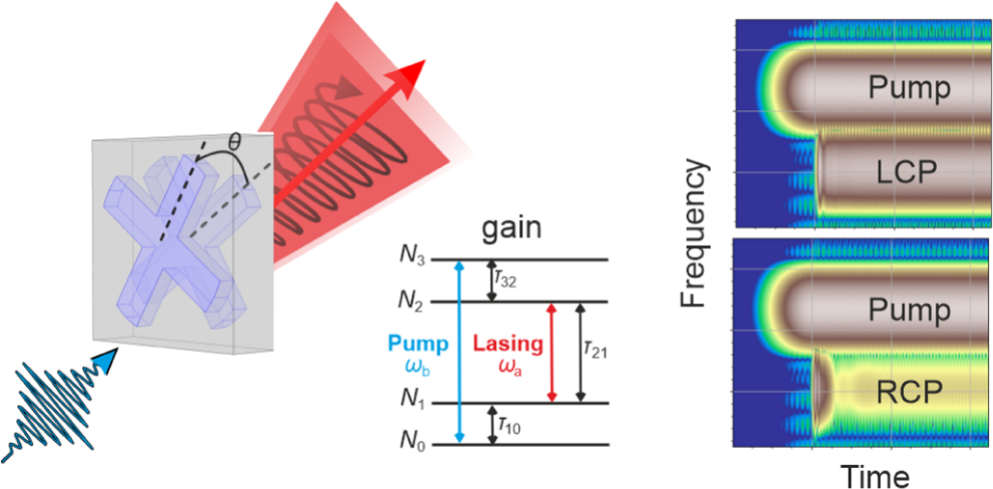

We demonstrate a simple, low-cost, and ultracompact chiral resonant metasurface design, which, by strong local coupling to a quantum gain medium (quantum emitters), allows to implement an ultrathin metasurface laser, capable of generating tunable circularly polarized coherent lasing output. According to our detailed numerical investigations, the lasing emission can be transformed from linear to circular and switch from right- to left-handed circularly polarized (CP) not only by altering the metasurface chiral response but also by changing the polarization of a linearly polarized pump wave, thus enabling dynamic lasing-polarization control. Given the increasing interest for CP laser emission, our chiral metasurface laser design proves to be a versatile yet straightforward strategy to generate a strong and tailored CP emission laser, promising great potential for future applications in both photonics and materials science.

## Introduction

Lasers with controllable, circularly polarized (CP) output hold significant promise for spectroscopy, sensing, and display technologies, and constitute a growing area in the field of light-matter interactions.^1−9^ In particular, CP emission lasers can serve as valuable tools for driving and investigating chiral-light-matter interactions, playing a critical role in various scientific disciplines, such as chemistry,^10^ biophysics,^11^ and quantum optics.^12−15^ Fundamentally, achieving CP lasing requires a combination of quantum gain and chiral response.^16^ One avenue is to use natural chiral molecules^17^ combined with a gain medium.^18^ However, this strategy is inefficient due to the very weak chiral response of the molecules. Another option uses chiral nematic liquid crystals combined with active (gain) molecules,^19−21^ but the synthesis of systems that simultaneously maintain both gain and chiral nematicity is not easy or efficient either. Recently, chiral light sources based on two-dimensional transition metal dichalcogenides (TMDCs)^22−25^ and perovskites^26−30^ combined with metamaterials have attracted attention due to their ability to give chiral photoluminescence. Nevertheless, the degree of circular polarization demonstrated in such media remains weak, limiting their potential for practical applications. An alternative to the chiral “molecules” approach is to employ chiral nanophotonic structures (operating as optical cavities). In recent years, various nanophotonics structures have been proposed to modulate the environment of the quantum emitters and to enhance the radiation emission or/and control its polarization state.^31−42^ Among these, chiral metasurfaces based on quasi-bound states in the continuum^43^ are remarkable because they can generate purely CP laser output. However, they do not offer dynamic polarization control and are not rigorously planar, which complicates their fabrication. Therefore, a flexible strategy for a controllable CP laser at large scale with low-cost and simple fabrication remains a challenge. Here, we propose an approach based on a compact, chiral resonant bilayer metallic (plasmonic) metasurface, locally strongly coupled to a thin, realistic quantum gain medium, and demonstrate its potential for controllable CP lasing.

Chiral metamaterials and metasurfaces (CMMs) are artificial, periodic arrangements of subwavelength resonant scatterers with no mirror symmetry plane^44−49^ and characterized by strong chiral magneto-electric coupling. This strong chirality is possible because their response is typically resonant and not restricted by the atomic size as in natural chiral materials, resulting in strong circular dichroism and large polarization rotation at arbitrary frequencies, as has been demonstrated in a variety of theoretical and experimental studies.^49−51^ Additional polarization control possibilities have been demonstrated by combining chiral metamaterials and metasurfaces with gain media in a Parity-Time symmetric configuration.^52−54^

Prompted by these developments, here we demonstrate that a resonant plasmonic biisotropic chiral metasurface, locally strongly coupled to a realistic quantum gain medium, constitutes a promising approach to ultracompact controllable CP emission laser. In such a metasurface, strong coupling to the quantum gain material mediated by the resonant near-field of the chiral meta-atoms will compensate for the losses in the plasmonic resonators and eventually have them spontaneously oscillate coherently, i.e., drive them into the laser state. The lasing output of this metasurface laser is the CP radiated field of the lasing resonant plasmonic eigenmode of the chiral metasurface, where the chiral resonant meta-atoms effectively constitute the subwavelength, plasmonic “resonant cavity” of the laser, allowing for strongly enhanced light-matter interaction and subwavelength size. (Note that for properly designed chiral metasurfaces the scattered field can be made purely CP.)

For the chiral metasurface considered here, we employ a design based on two mutually twisted metallic crosses (as, e.g., in ref. (55)) and validate the polarization controllable lasing by numerical simulations. Adjusting the twist-angle between the crosses or, more importantly, adjusting dynamically the pump polarization angle enables the manipulation of the chiral response, which couples with the optical gain band, thus allowing different laser emission behaviors to be demonstrated. Our active (gain) chiral metasurface provides an opportunity to design lasing with any desired polarization state, including CP, eliminating the need for expensive and tedious fabrication. The twisted-cross design as the chiral resonator fundamentally allows for comparatively easy planar layer-by-layer construction despite creating a chiral, three-dimensional nanostructure. Although beyond the scope of this article and our experimental capabilities, we envision several possible routes for fabrication of the proposed chiral lasing system, which are described in the Supporting Information.

## Model and Physical Mechanism

**Figure 1:**
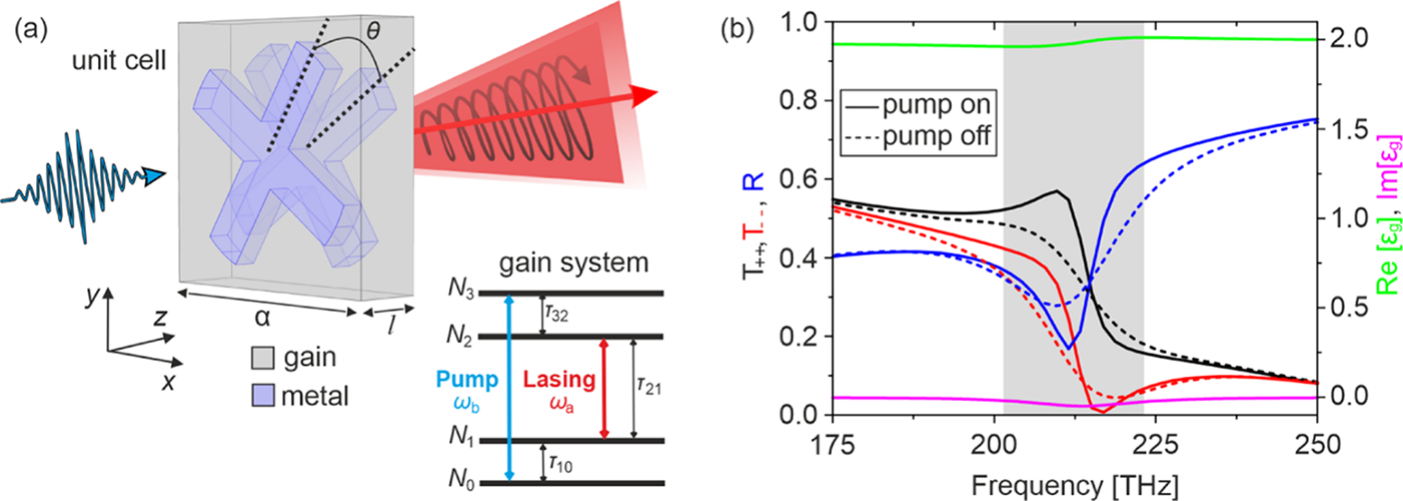
(a) Schematic of unit cell and four-level gain system of our chiral metasurface. (b) Corresponding calculated linear scattering spectra. The solid (dashed) black, red, and blue lines show the circular polarized diagonal transmission and reflection amplitudes with (and without) a pump, respectively. The green and magenta solid lines show the real and imaginary part of the permittivity of the gain medium, respectively, corresponding to eq (7), at a pump rate see eq (7), at pump rate  s^–1^. The shaded region in the background indicates the gain material bandwidth.

**Figure 2:**
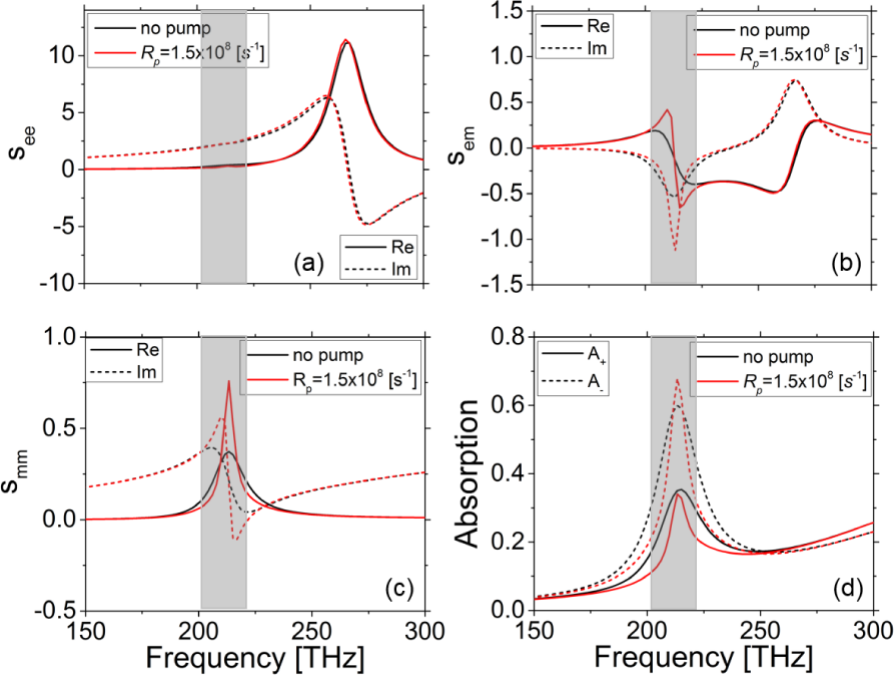
Real (solid lines) and imaginary (dashed lines) parts of the dimensionless effective electric (a), magneto-electric (b), and magnetic (c) sheet conductivities for the system of Figure 1, at pump rates  and . Panel (d) shows the corresponding absorptions for RCP/+ and LCP/– waves. The shadow indicates the gain bandwidth.

The unit cell of our chiral metasurface is shown in Figure 1a. It consists of two mutually twisted metallic crosses of twist angle  (the first cross is rotated 11.25^◦^ to the left with respect to the diagonal while the second 11.25^◦^ to the right), embedded in a dielectric host of refractive index *n* = 1.41. The metasurface has square periodicity (along  directions) with lattice constant *a* = 300 nm, and thickness *l* = 100 nm. The metallic crosses are made of silver, described by a Drude model  with ,  rad/s, and  rad/s. Geometric parameters of the crosses are their height (side-length), *h* = 250 nm, width, *w* = 56 nm, and metal-thickness, *s* = 25 nm. Figure 1b illustrates the calculated CP linear scattering spectrum (with and without gain) of transmittances *T*_++_ and *T*_–_ and reflectances  for the structure of Figure 1a. The first (second) subscript denotes the output (input) wave polarization, with + and – indicating right-handed (RCP) and left-handed (LCP) circularly polarized wave, respectively. Note that the cross-polarized transmittances and copolarized reflectances are zero (see the Supporting Information). Figure 1b shows a resonance around 214 THz, which is a predominantly magnetic resonance rendered chiral by the twist between the crosses (with antiparallel currents at the two “facing” crosses—see the Supporting Information; also ref.^56^). This local magnetic resonance is strongly coupled to the gain/substrate material via the near field, leading to reshaping and undamping of the resonance as depicted in Figure 1b and explained in more detail below (see also Figure 2).

Note that in the linear scattering simulations, the contrast of transmittances for RCP and LCP waves is not very large for small gain. However, when pumped with gain strong enough to cause spontaneous oscillation (i.e., lasing of the resonant mode), one chiral mode actually “survives”, while the other is suppressed with significant enhancement of the contrast. This is apparent from Figure 4 in the manuscript. Despite the moderate contrast in the linear transmission amplitudes of the chiral resonators without or with little gain (see Figure 1b), if strongly pumped, the lasing system settles into a rather pure LCP or RCP lasing emission. After some initial brief excitation of both modes, one of the CP lasing modes becomes the dominant lasing mode at the expense of the other, which is suppressed and quickly dies out.

The gain material can be obtained by doping the dielectric host with dyes, described here as four-level systems, as depicted in Figure 1.^57−61^*N*_*i*_ is the population density in each level . Initially all the electrons are in the ground state . Then, electrons are pumped by an external electromagnetic wave with frequency , where *E*_3_, *E*_0_ are the energies of the excited (third level) and ground state (zero level), respectively. After a short lifetime τ_32_, the electrons relax into metastable level 2. By spontaneous and stimulated emission, as well as by nonradiative processes, the electrons are transferred to level 1. Levels 1 and 2 are the lasing states and the lasing frequency is . The lifetimes and energies of the lasing levels are τ_21_, *E*_2_ and τ_10_, *E*_1_. The atomic populations at each spatial point obey the following rate:^57−61^
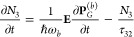
1
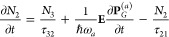
2
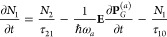
3
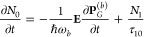
4where  is the induced electric polarization density of the atomic transition between the lasing levels (1 and 2, radiation emission),  is the induced electric polarization density of the transition between ground and excited state (0 and 3, pumping, radiation absorption), and the dots on the populations and polarizations indicate time derivative. The induced macroscopic polarization is related with the microscopic polarization of the molecules (see the Supporting Information) and is coupled to the local electric field **E** via the following equations:
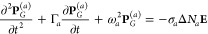
5
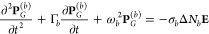
6where , ; ,  are the line widths of the two optical electronic transitions, and σ_*a*_, σ_*b*_ are the coupling strengths of ,  to the electric field **E**, the values of which can be obtained experimentally. The Maxwell’s equations are coupled with the rate eqs 1–4 via eqs 5 and 6, through the polarization density of the gain system, while all equations share the same simulation domain, along with the same spatial and temporal discretization. We integrate our four-level formalism into the chiral metasurface and perform the combined system analysis using Finite Element Time Domain (FETD) simulations through COMSOL Multiphysics software. The parameters for the four-level system are chosen as follows:^57,62^ Total electron density  m^–3^, coupling coefficients  C^2^/kg,  C^2^/kg, line widths  rad/s,  rad/s and relaxation times  ps,  ps.

## Results and Discussions

### Scattering Simulations and Retrieval Procedure

In order to better understand the population dynamics and the effective polarizablility (permittivity) contributed by the gain system, we consider the stationary state lasing, replacing the pump field by an average pumping rate, i.e., approximating the rate of absorbed pump photons per volume, , by an abstract pumping rate of electrons from the ground level to the top level, . The pump rate *R*_*p*_ is related to the absorbed pump intensity, *I*_*pump*_, via , where *l* is the thickness of the gain layer within the metasurface. Then the polarization density induced by the gain medium can be incorporated in the frequency-dependent constitutive relations, , where *ε*_*h*_ is the host relative permittivity. Applying the steady state approximation assuming weak fields in eqs 1–5), neglecting all nonlinear terms except the leading order (which is constant in the population numbers, , and linear harmonic time-dependence in the electric field and polarizations, we can express the relative permittivity of the gain-host material as^62−64^
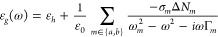
7where , , and . Under the chosen conditions, we find from eq 7 that the contribution from the lasing transition is about 1 order of magnitude smaller than the host permittivity *ε*_*h*_, which will affect the metasurface by renormalzing the resonances of the chiral meta-atoms, both in frequency and damping, while the correction from the pump transition is yet another order of magnitude weaker and mostly negligible except for the fact that it renders the lasing resonance of the chiral metasurface slightly sensitive to the intensity (and polarization) of the pump radiation. For the metasurface shown in Figure 1a and pump rate 1.5 × 10^8^ s^–1^ the permittivity  is shown in Figure 1b, together with the corresponding metamaterial scattering amplitudes. Note that the coupling of the chiral structure with the gain material results in undamping of the metamaterial resonance, as is expected (see also ref. (65)). This change is highly affected by the pump rate, *R*_*p*_.

To quantify the chiral structure response and examine how it is affected by the gain we use the standard retrieval procedure^66^ suitable for thin metamaterial layers/sheets, to extract the effective dimensionless sheet conductivities, *s*_*ee*_, *s*_*mm*_, *s*_*em*_, of the structure with and without gain (see also effective material parameters in the Supporting Information). Figure 2a shows the retrieved results for the real (solid lines) and the imaginary (dashed lines) parts of the effective electric conductivity *s*_*ee*_, with gain and without gain. One can see that increasing the pump rate and hence the available gain leads to a slight decrease in the real part of the electric dimensionless conductivity in the frequency range close to the maximum emission cross-section of the gain medium, demonstrating reduced losses (in agreement with Figure 1b). Figure 2b shows the real (solid lines) and imaginary (dash lines) parts of the effective magnetic conductivity *s*_*mm*_, with and without gain. We observe that with the incorporation of gain, the weak and broad magnetic resonance of the passive metasurface becomes strong and narrower, indicating that the electric gain counteracts the losses in the magnetic/chiral resonance. Besides effective electric and magnetic conductivities, we also calculate the effective sheet magneto-electric conductivity *s*_*em*_, a measure of the chiral response of the structure. Figure 2c illustrates the real and the imaginary parts of *s*_*em*_, with and without gain. We see that increasing the pump rate leads to a strong enhancement of both real and imaginary parts of *s*_*em*_, i.e., a strong enhancement of the system chiral response (see the Supporting Information for details).

In order to clarify and quantify the impact of gain on the polarization state of the transmitted wave through the system, we calculate the absorption for RCP (+) and LCP (−) waves (determining the wave ellipticity; for the corresponding ellipticity see Supporting Information)—see Figure 2d. One can see that the increase of pump rate reduces the absorption peak for LCP waves, while it narrows the peak for RCP waves without affecting much its maximum value. The corresponding ellipticity, which without gain is close to 20^◦^, for  s^–1^ gets close to 40^◦^, indicating pure circularly polarized waves. Hence, we find that the effective amplification in the gain-metasurface system, which is controllable via the pump rate, highly affects the polarization state of the output wave, allowing dynamic polarization tuning. It is worth-noting here that the LCP absorption (*A*_–_) is more sensitive to the gain value; as we will show later, it is this mode (LCP), which will first lead to lasing.

### Circularly Polarized Lasing

In the above analysis, we show the strong coupling between gain and chiral response, resulting in gain-controllable transmitted wave ellipticity; however, the main objective of this study is to demonstrate controllable (including circular) polarization lasing in our structure. For this demonstration and the understanding of the underlying physical mechanisms, we examine the active chiral metasurface of Figure 1a employing the “quantum” approach for the gain, i.e., eqs 1–5, and linearly polarized input/pump wave.

**Figure 3:**
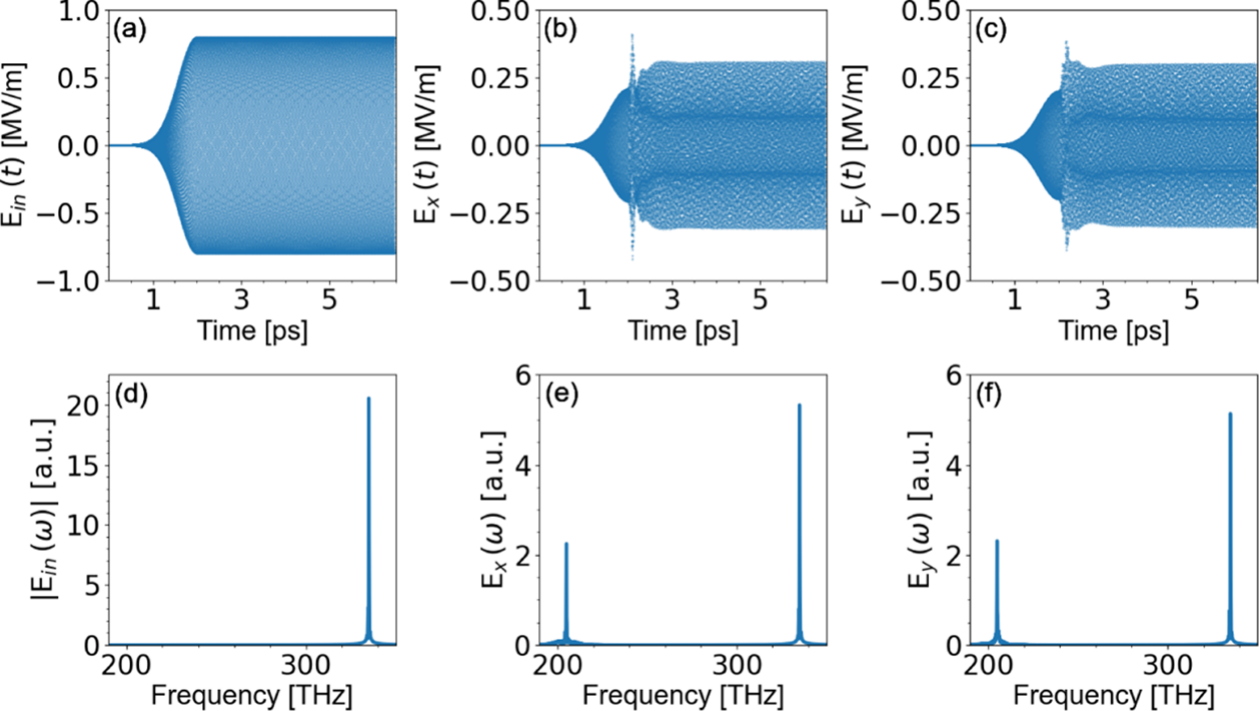
Incident and transmitted waves as a function of time for the structure of Figure 1 excited by a continuous wave (CW) of frequency 335 THz and polarization angle . Panels (a), (b), and (c) are, respectively, the incident, transmitted *E*_*x*_, and transmitted *E*_*y*_ waves for input pump amplitude  V/m (for the incident wave we show the component along the *x*–*y* diagonal). Panels (d), (e), and (f) are the corresponding Fourier transformed spectra of the incident and transmitted waves. The sharp peak at about 335 THz is the incident beam (driving the gain from level 0 to 3).

We pump the gain molecules with a continuous wave (CW) pump of frequency  rad/s; . Figure 3a–c depicts the incident, copolarized transmitted, and cross-polarized transmitted waves as a function of time for polarization angle . Then, we Fourier transform the time-dependent transmitted electric fields to see if there is emission and how strong is the emitted radiation around  rad/s (lasing frequency). The results are depicted in Figure 3d–f. The pump amplitude, *E*_*p*_, is  V/m and we do observe a peak at the emission frequency. An even higher amplitude of the pump wave leads to a higher emitted/lasing power. As shown in Figure 3 the *x* and *y* components of the lasing field have equal amplitudes. Observing their relative phase we see that it is close to . This shows that the lasing wave is a wave of circular polarization

**Figure 4:**
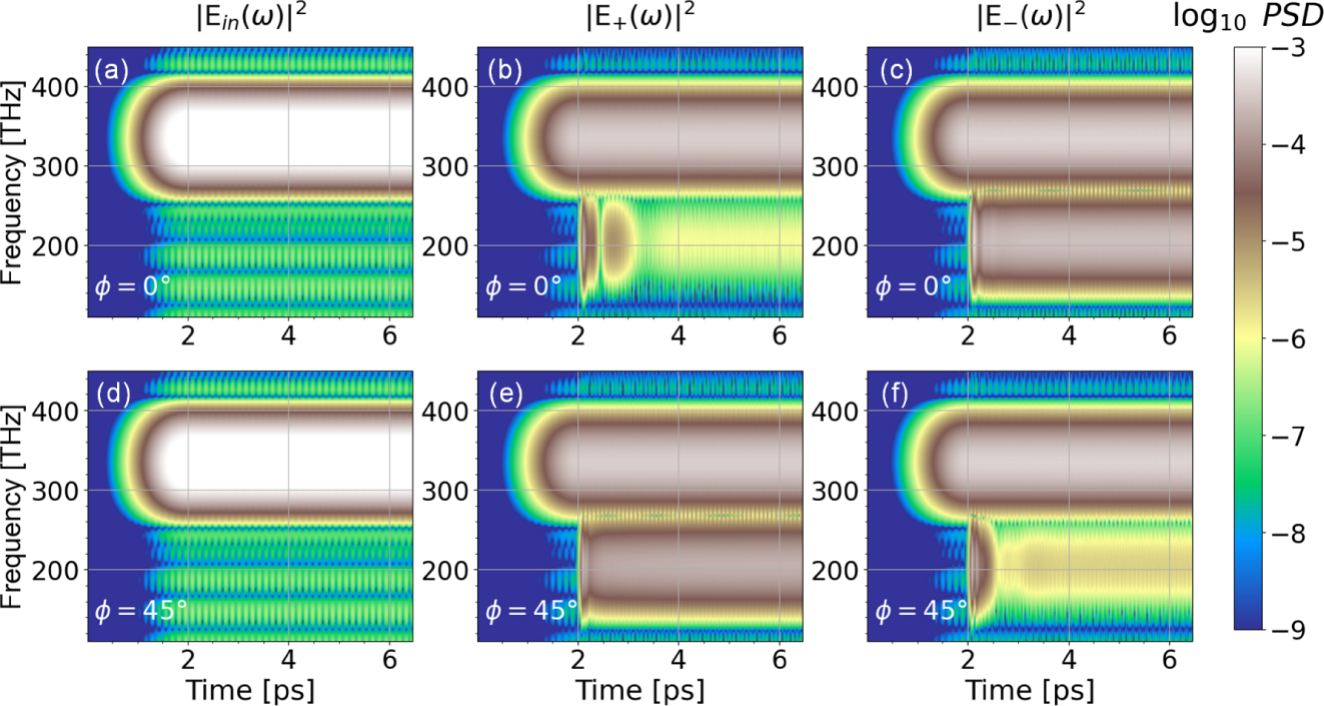
Incident and transmitted power spectral density (color) from the structure of Figure 1 for incidence of a linearly polarized CW of different polarization angles, ϕ. Panels (a), (b), and (c) show the incident, RCP transmitted, , and LCP transmitted, , wave, respectively, as a function of time and frequency for incident polarization angle . Panels (d), (e), and (f) show the corresponding results for incident pump polarization angle . For other angles, please see Figure S7 in the Supporting Information.

To further investigate the lasing wave polarization and examine the possibility of its control, we keep the same pump amplitude,  V/m and modify the relative polarization angle, ϕ, from , corresponding to a linearly polarized incident pulse along the *x*-axis, to , corresponding to linearly polarized incident pulse along *y*-axis. The results for  and  are shown in Figure 4, where the two rows denote the two different angle cases, and both input and output waves are transformed in the circular polarization basis. Figure 4a,d shows the power spectral density (in log scale) of the incident, Figure 4b,e of the RCP transmitted (*E*_+_), and Figure 4c,f of the LCP transmitted (*E*_–_) waves. When the polarization angle is  we observe that above 2.5 ps the *E*_+_ polarized emitted wave vanishes, while the *E*_–_ polarized wave is the dominant, indicating the pure circularly polarized output. As we tune the polarization angle to , we observe an interchange between the *E*_+_ and *E*_–_ transmitted waves at the lasing frequency; the dominant transmitted wave is now the RCP, *E*_+_, wave. (Further increase of the polarization angle, i.e., , leads again to LCP transmitted output; intermediate cases are shown in the Supporting Information.) Defining the purity of the circularly polarized output as the ratio of the intensity of the desired circular mode and the total power radiated, we find for the two lasing modes shown in Figure 4: for RCP  and for LCP . This indicates that by modifying the angle ϕ one can dynamically tune the polarization state of the lasing mode, going from linear to circular polarization and from RCP to LCP, a capability of high importance in all applications based on or affected by the wave polarization.

**Figure 5:**
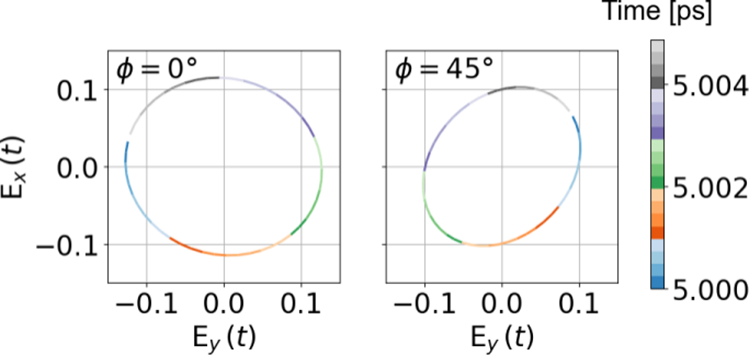
Transmitted waves *E*_*x*_ versus *E*_*y*_ for different polarization angles of the incident wave,  and  (see legends), for a specific time duration, denoted in the colorbar, constituting one wave period.

Note that Figures 3a–c and 4 show the time domain and the time–frequency domain spectrogram of the lasing output, respectively, after the pump is switched on. We clearly see the laser settling into an almost purely circular polarized stationary output after some brief fluctuations. The peaks in the Fourier power spectra shown in Figure 3d–f indicate the pump and lasing frequencies in the stationary limit for pump radiation and output radiation in linear (x, y) polarizations, corresponding to the “beating pattern” of linear scattering of the pump and lasing radiation observed in Figure 3b,c, and motivate the frequency range shown in the spectrogram in Figure 4, albeit here in circular (+, −) polarizations to better identify the circular polarized nature of the lasing output. Finally, Figure 5 shows the output E-field vector in the time domain over a single period in the stationary state, directly confirming the circular polarized nature of the lasing output.

To confirm and investigate further the polarization and the handedness of the outgoing laser radiation, we also calculate the linear components of the transmitted waves, *E*_*y*_ versus *E*_*x*_, for a specific time duration. The results over a single period are listed in Figure 5. We observe that at , an almost perfect circularly polarized emission laser is achieved. The results indicated by the color in Figure 5 clearly show that at  we observe an anticlock (LCP) wave, while at  a clockwise wave (RCP). The above analysis clearly shows that our structure and approach can lead to a controllable and pure circular polarization laser. To the best of our knowledge, up to date, there are no efficient routes to demonstrate and control pure (with a high degree of circular polarization) CP lasing in an ultracompact form.

Finally, we highlight here an unexpected feature observed by comparing the results of Figures 2d and 5. We observe that the lasing mode in Figure 5 (LCP mode, left panel) is associated with higher absorption and thus lower transmittance below the lasing threshold compared to the other CP mode. In other words, while our chiral structure, in the absence of gain, shows large cicrular dichroism resulting in highly RCP transmittance, it first emits an LCP wave when lasing occurs. This is not surprising though, taking into account that emission is in fact the inverse of absorption, and both processes are determined by the same material characteristics. Thus, the mode of the higher absorption below lasing threshold is the one that first emits/lases above the lasing threshold.

## Conclusions and Outlook

In conclusion, we have presented a simple planar, ultracompact active chiral resonant metasurface design that allows easy realization of an ultrathin metasurface laser capable of generating circularly polarized coherent lasing output. This output arises from the direct lasing action of a collective resonant eigenmode of the periodic, resonant chiral meta-atoms of the metasurface. The meta-atoms are formed of a pair of metallic (plasmonic) crosses that are relatively twisted. Strong coupling to the quantum gain material, mediated by the resonant near-field of the meta-atoms, will compensate for the dissipative losses in the plasmonic meta-atoms and eventually lead them to oscillate coherently, i.e., drive them into a lasing state. The chiral resonators here effectively constitute the subwavelength “resonant cavity” of the laser, allowing for strongly enhanced light-matter interaction and subwavelength size. We have shown that both the geometrical twist-angle of the metallic crosses, as well as the polarization of an incident pump radiation, can be used to control the emission polarization state of the laser from linear to circular and to switch from the right- to left-circularly polarized output. We believe that our findings could guide new experimental efforts toward realization of polarization controllable circularly polarized output ultrathin surface lasers using resonant chiral metasurfaces, which offer access to a plethora of exciting photonic applications.

## Supporting Information Available

Scattering simulations; retrieval procedures; additional results verifying the dynamic lasing polarization control possibility; derivation of macroscopic polarization through microscopic polarization of molecules; simulation method(ZIP)
Detailed account of the physical and mathematical verification of the linear scattering response as well as the dynamic laser polarization control possibility (PDF)
Figures S1–S7(ZIP)
