# Supplementary figures and images for: Ultrathin, Dynamically Controllable Circularly Polarized Emission Laser Enabled by Resonant Chiral Metasurfaces
Source: ACS Photonics. 2024 Nov 22;12(1):71–8. doi: 10.1021/acsphotonics.4c01005 (PMC11741137; doi:10.1021/acsphotonics.4c01005)

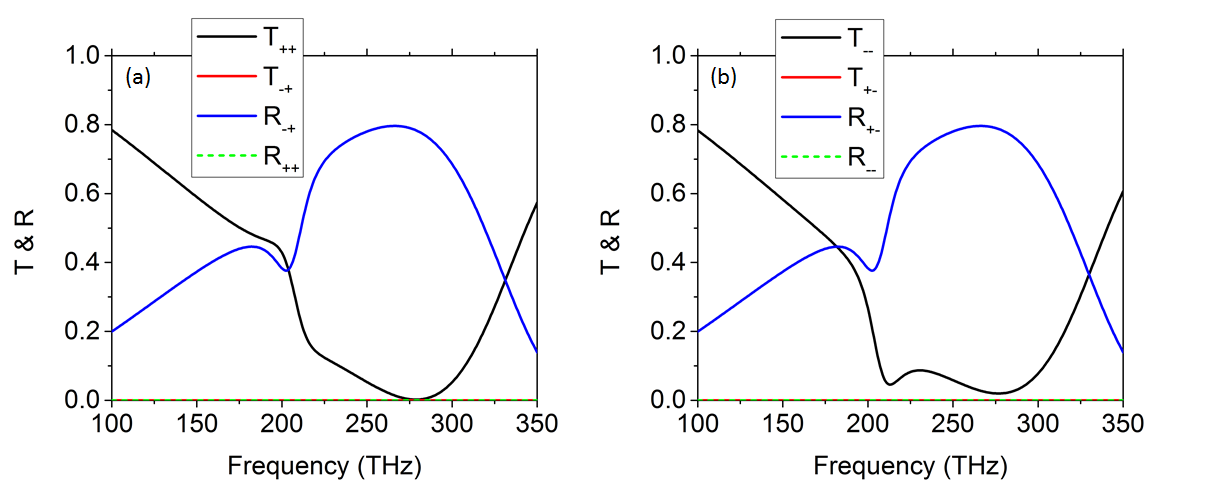

Supplement: Supplementary file 1 — ph4c01005_si_001.zip [file ph4c01005_si_001.zip › figS1.png]

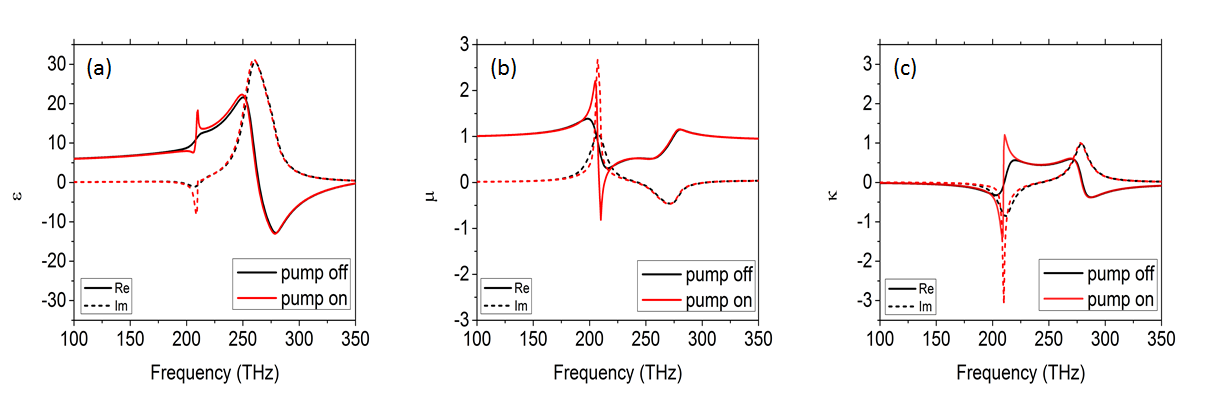

Supplement: Supplementary file 1 — ph4c01005_si_001.zip [file ph4c01005_si_001.zip › figS5.png]

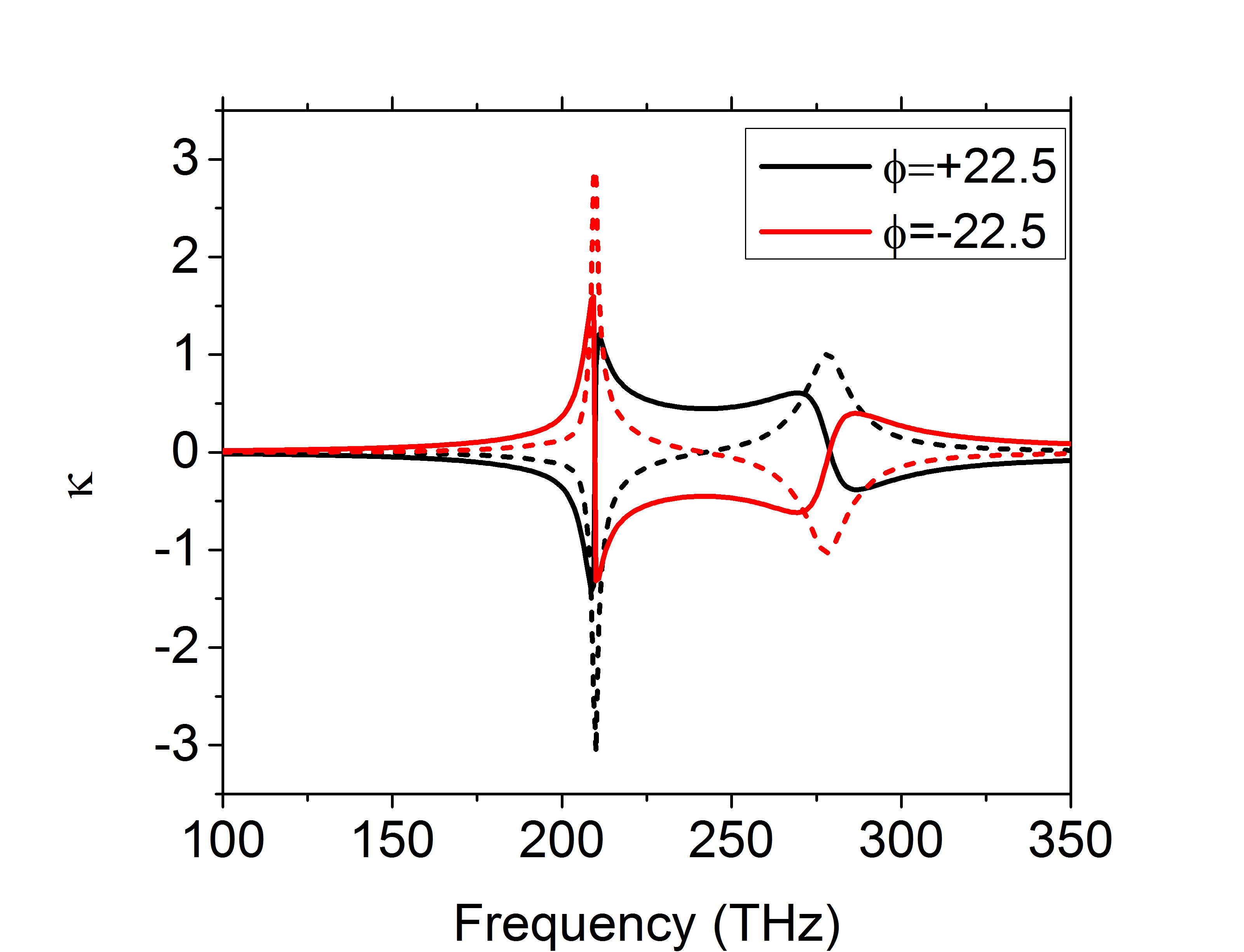

Supplement: Supplementary file 1 — ph4c01005_si_001.zip [file ph4c01005_si_001.zip › figS6.png]

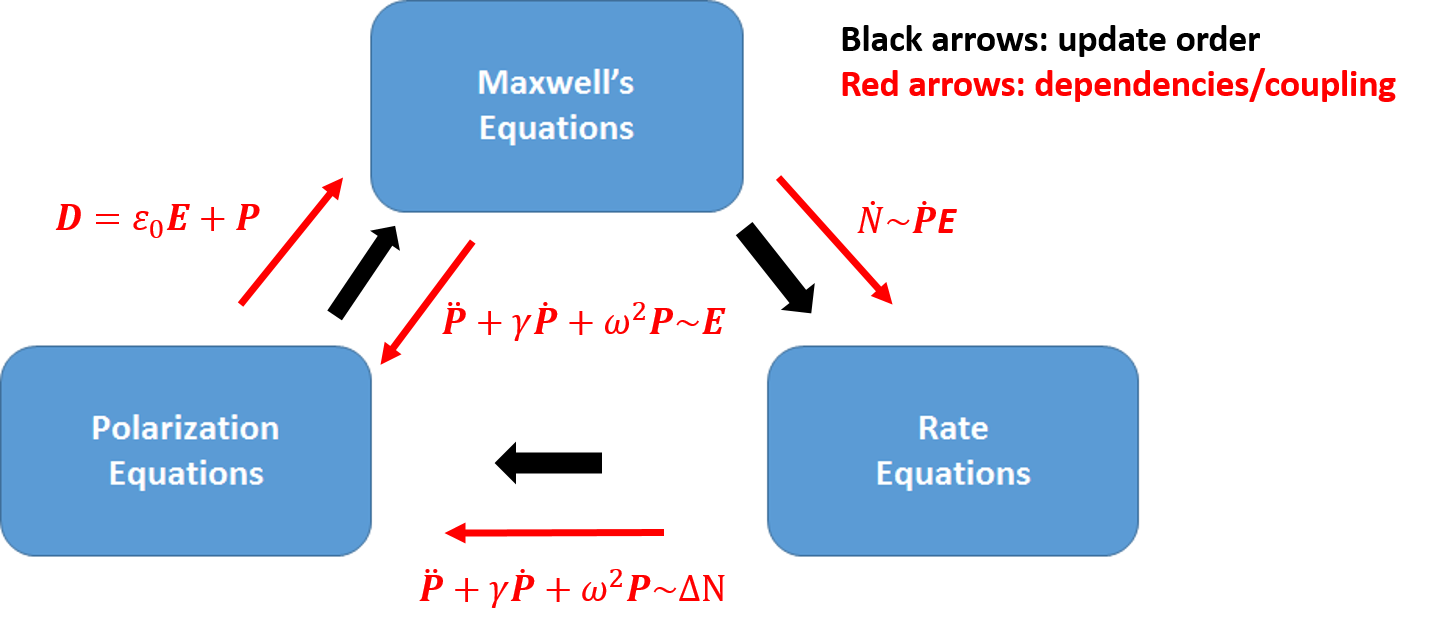

Supplement: Supplementary file 1 — ph4c01005_si_001.zip [file ph4c01005_si_001.zip › figS7.png]

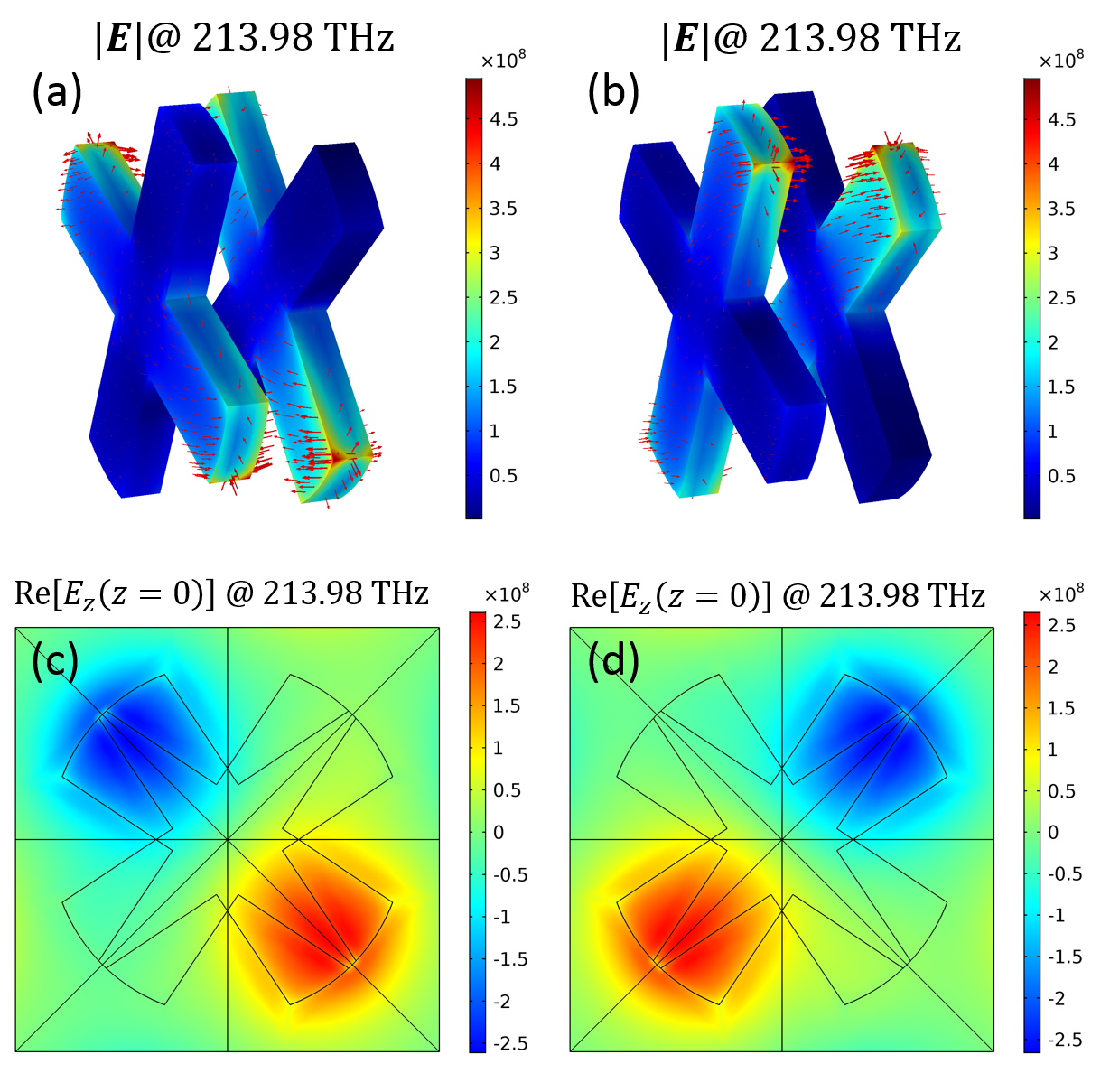

Supplement: Supplementary file 1 — ph4c01005_si_001.zip [file ph4c01005_si_001.zip › fig_S4.png]

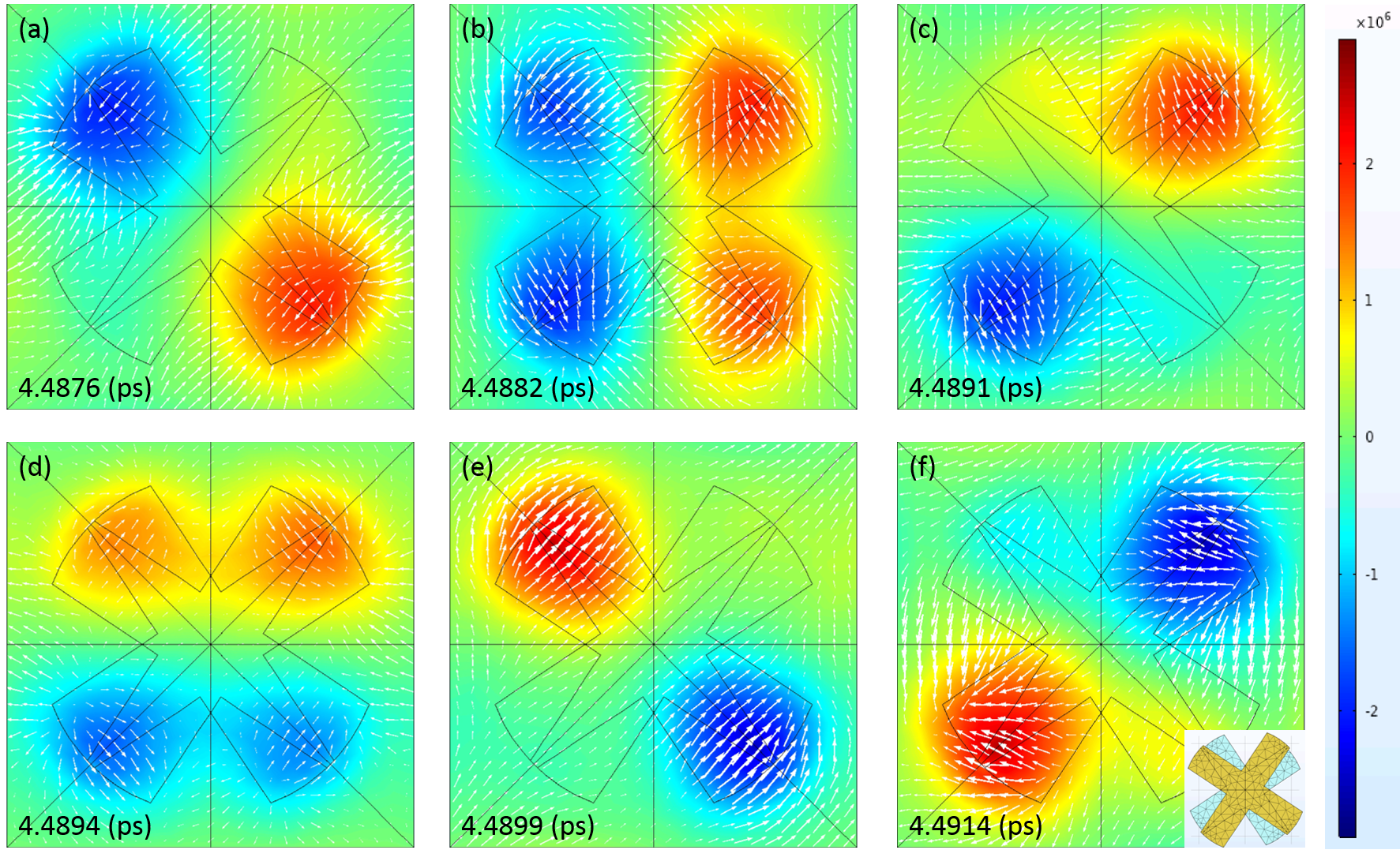

Supplement: Supplementary file 1 — ph4c01005_si_001.zip [file ph4c01005_si_001.zip › s1.png]

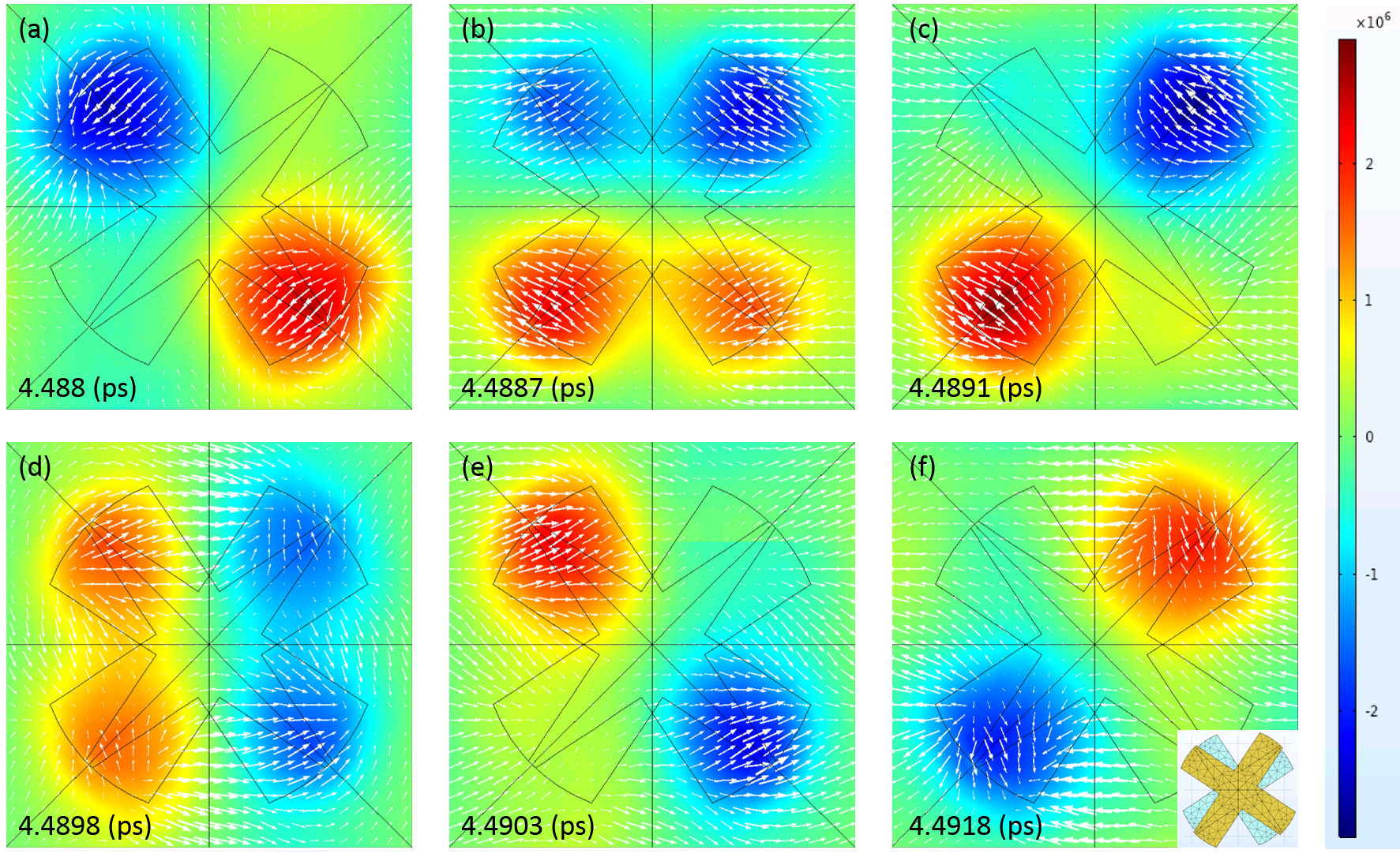

Supplement: Supplementary file 1 — ph4c01005_si_001.zip [file ph4c01005_si_001.zip › s2.png]

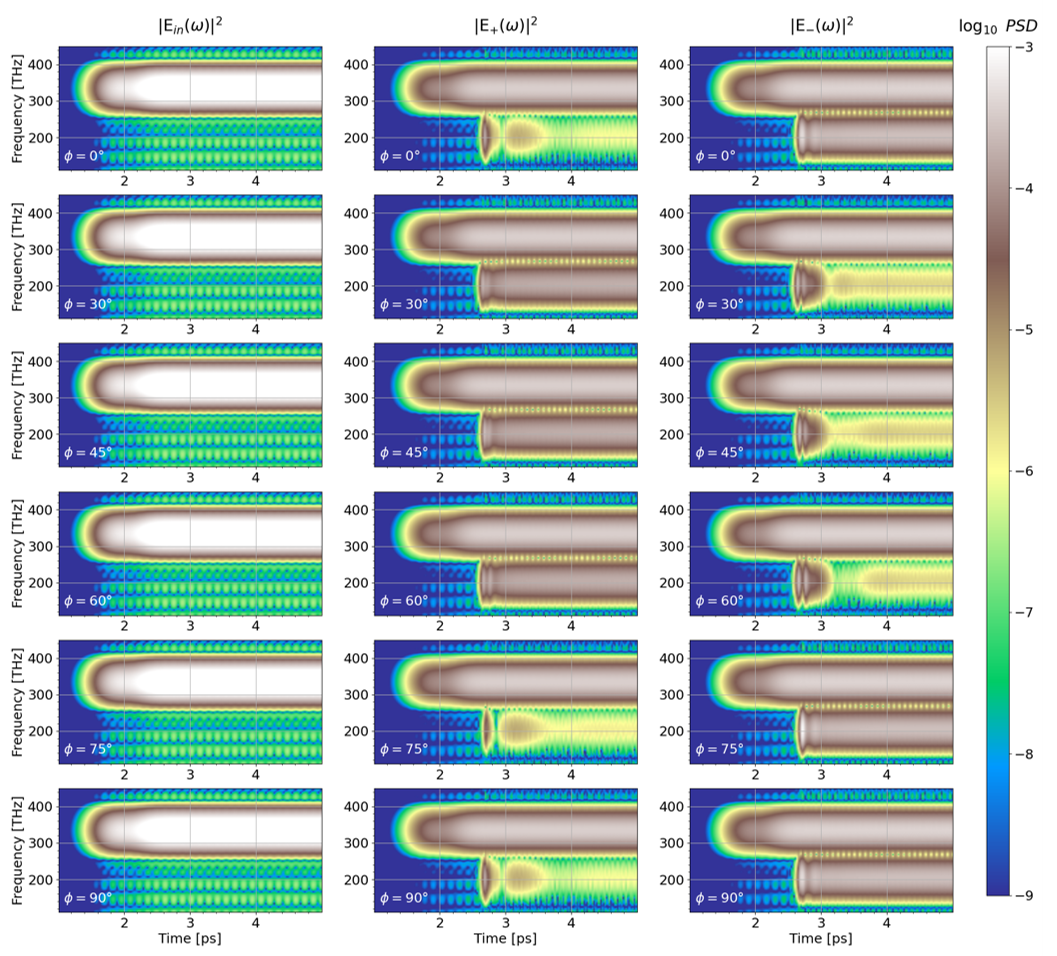

Supplement: Supplementary file 1 — ph4c01005_si_001.zip [file ph4c01005_si_001.zip › f5_paper.png]

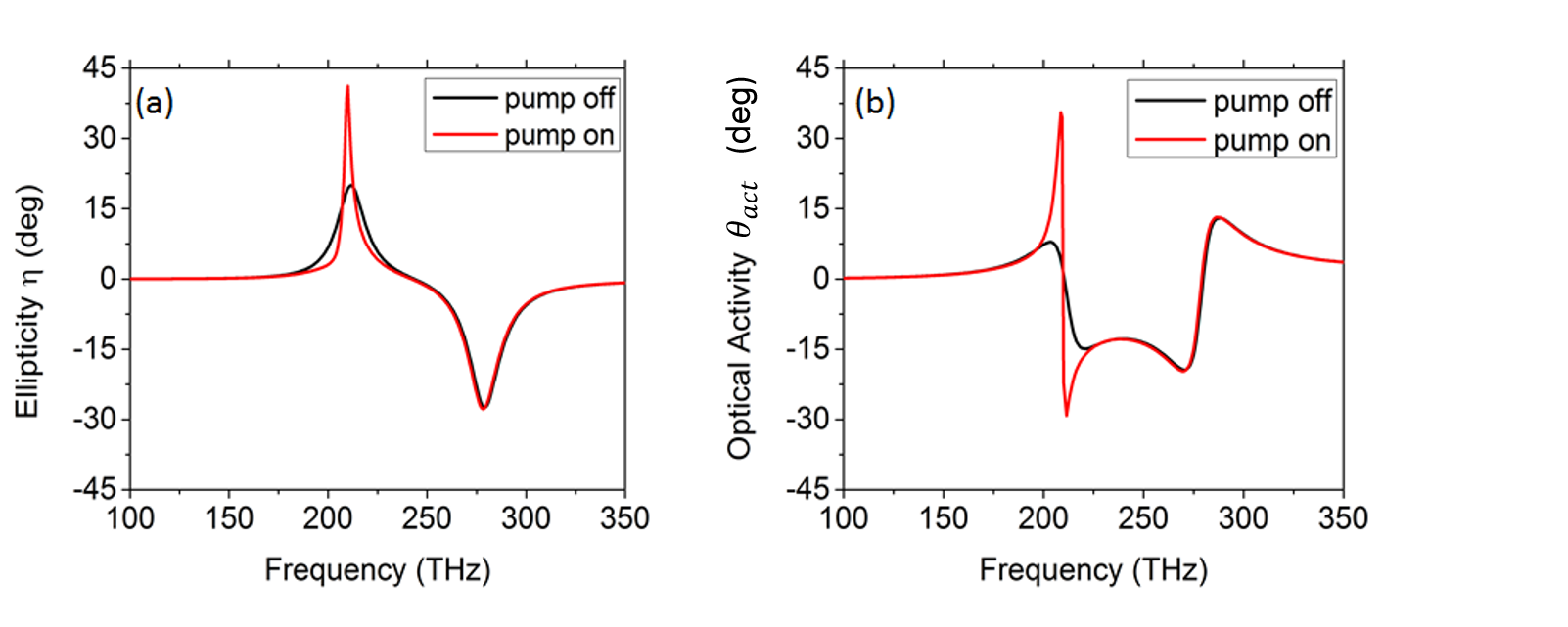

Supplement: Supplementary file 1 — ph4c01005_si_001.zip [file ph4c01005_si_001.zip › figS2IInew.png]

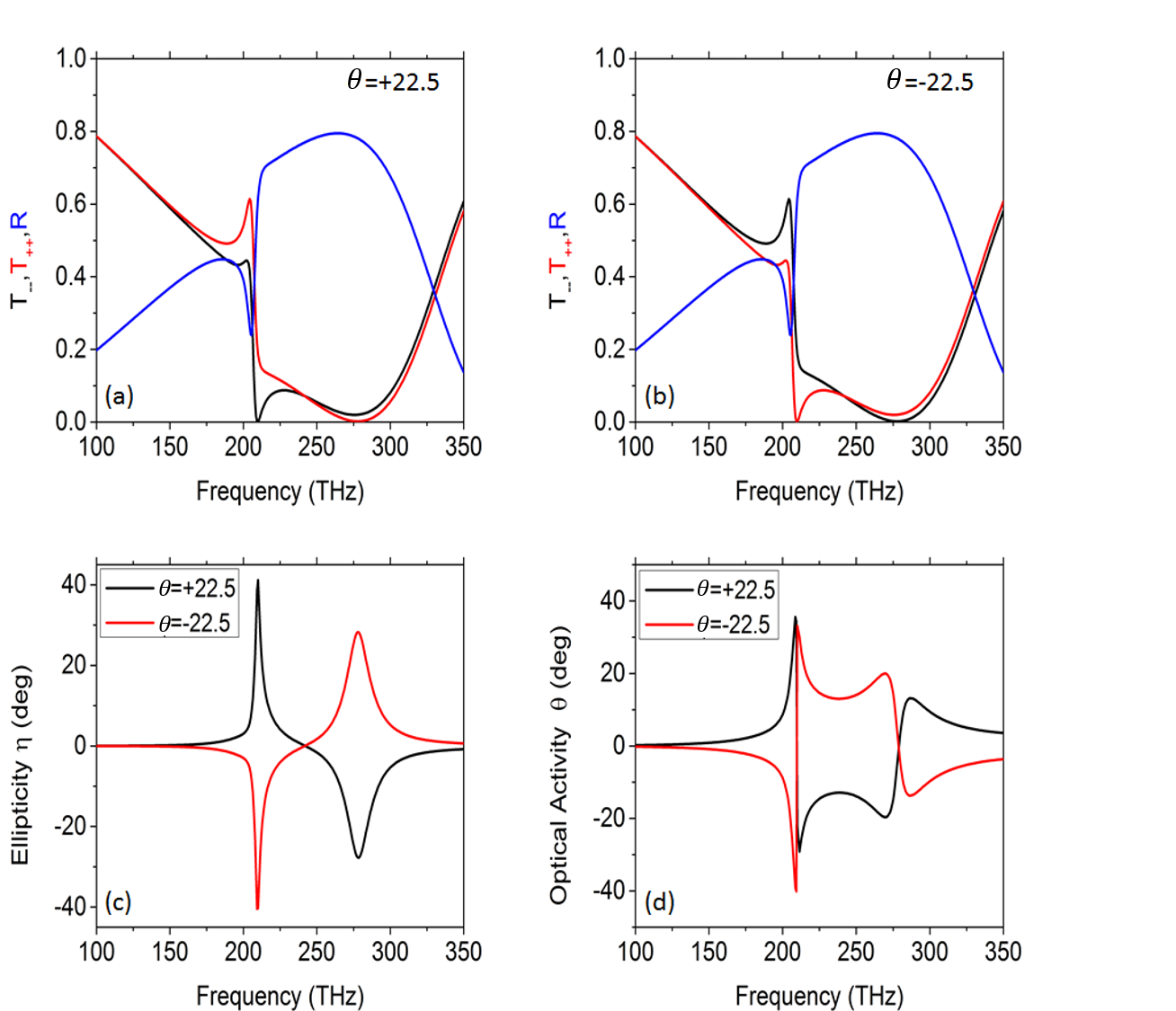

Supplement: Supplementary file 1 — ph4c01005_si_001.zip [file ph4c01005_si_001.zip › figS3new.png]

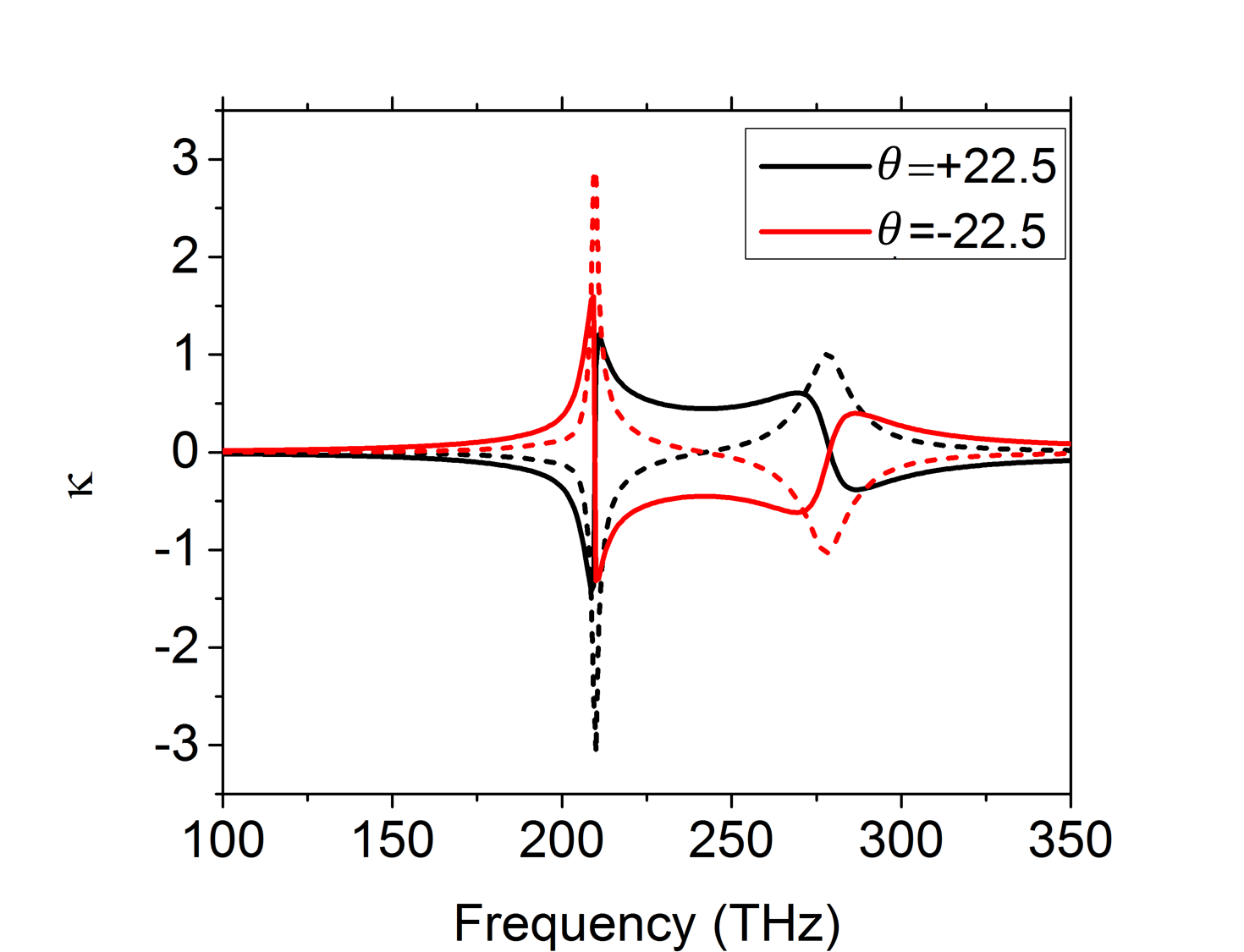

Supplement: Supplementary file 1 — ph4c01005_si_001.zip [file ph4c01005_si_001.zip › figS6new.png]

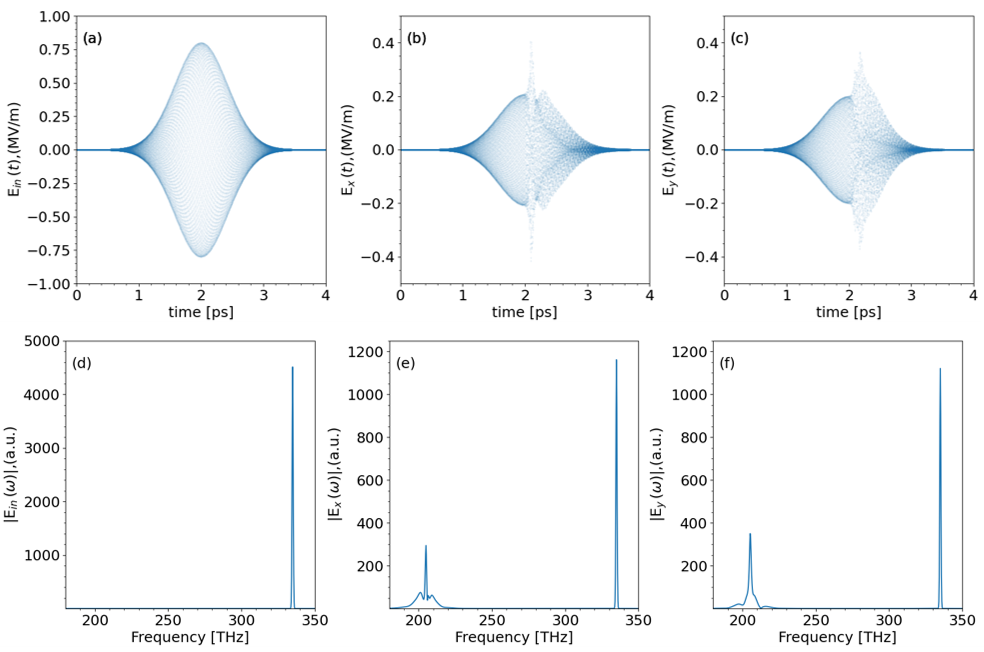

Supplement: Supplementary file 1 — ph4c01005_si_001.zip [file ph4c01005_si_001.zip › fig_3_paper_new_sup.png]

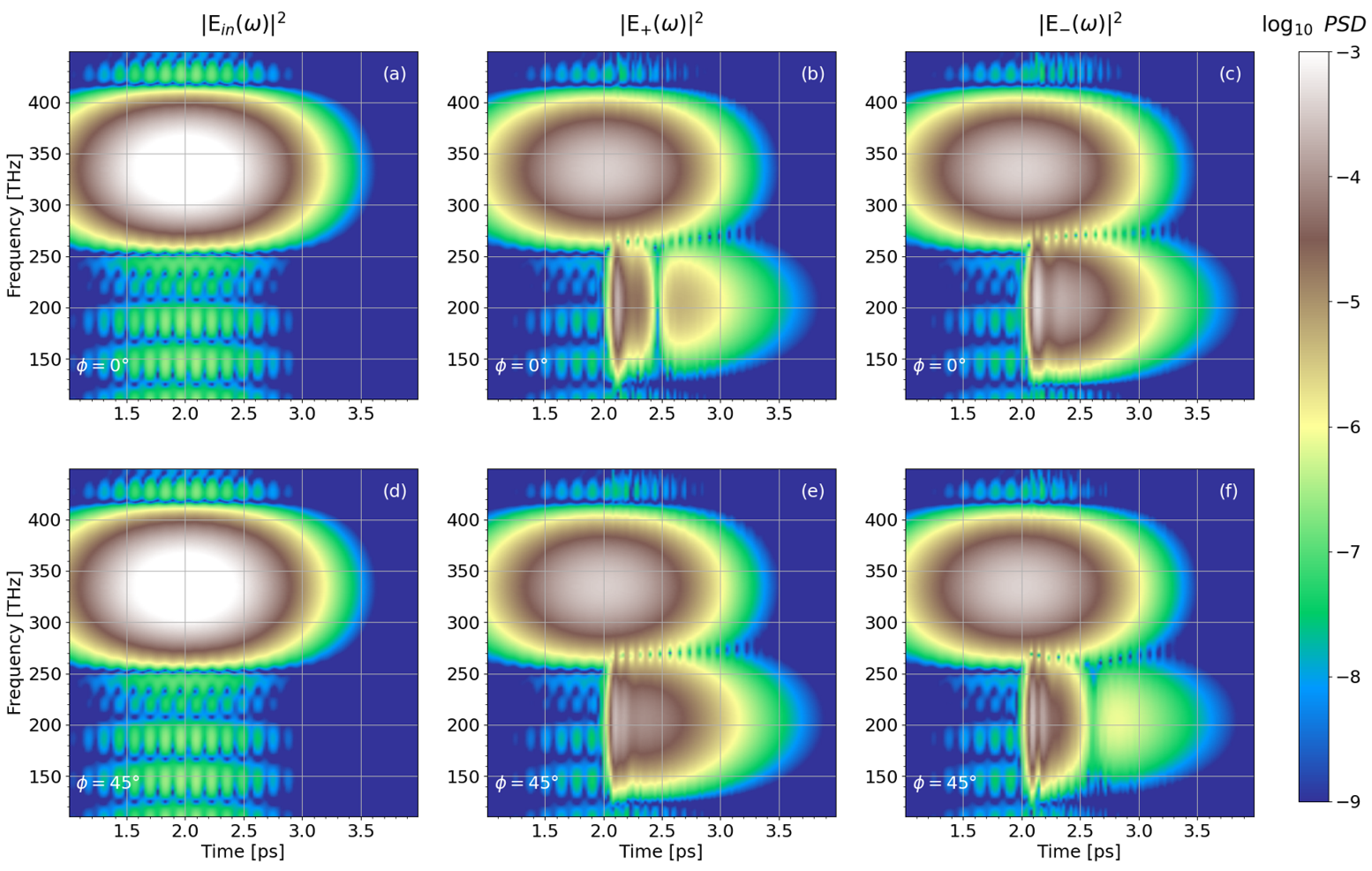

Supplement: Supplementary file 1 — ph4c01005_si_001.zip [file ph4c01005_si_001.zip › f4paper_new.png]
