# Supplementary material for: Ultrathin, Dynamically Controllable Circularly Polarized Emission Laser Enabled by Resonant Chiral Metasurfaces
Source: ACS Photonics. 2024 Nov 22;12(1):71–8. doi: 10.1021/acsphotonics.4c01005 (PMC11741137; doi:10.1021/acsphotonics.4c01005)
Supplement: Supplementary file 2 — ph4c01005_si_002.pdf [file ph4c01005_si_002.pdf]

Ultrathin, dynamically controllable circularly polarized emission laser enabled by resonant chiral metasurfaces

Ioannis Katsantonis, Anna C. Tasolamprou, Eleftherios N. Economou, Thomas Koschny and Maria Kafesaki

June 12, 2024

S1 Scattering simulations

Scattering simulations provide a complete description of the electromagnetic wave transmission and reflection by a structure. For chiral structures, where the eigenwaves are the circularly polarized waves, the scattering problem is usually formulated for circularly polarized light. To demonstrate the chiral response of our structure, we consider a unit cell as the one shown in Fig. 1(a) in the main text (without taking into account the gain response), with periodic boundary conditions along x and y directions, and calculate the reflected and transmitted fields for normally incident circularly polarized (CP) waves using commercial software COMSOL Multiphysics. In that case the incident (in), reflected (ref) and transmitted (tr) fields are related as

$$\begin{bmatrix} E_+^{(ref)} \\ E_-^{(ref)} \end{bmatrix} = \begin{bmatrix} r_{++} & r_{+-} \\ r_{-+} & r_{--} \end{bmatrix} \begin{bmatrix} E_+^{(in)} \\ E_-^{(in)} \end{bmatrix}, \tag{S1}$$

and

$$\begin{bmatrix} E_+^{(tr)} \\ E_-^{(tr)} \end{bmatrix} = \begin{bmatrix} t_{++} & t_{+-} \\ t_{-+} & t_{--} \end{bmatrix} \begin{bmatrix} E_+^{(in)} \\ E_-^{(in)} \end{bmatrix}. \tag{S2}$$

In Eqs. (S1)-(S2), $r_{++}, r_{+-}, r_{-+}, r_{--}$ and $t_{++}, t_{+-}, t_{-+}, t_{--}$ are the complex reflection and transmission amplitudes, where the first subscript indicates the output wave polarization and the second the incident wave polarization, while the subscripts +, - indicate right-handed and left-handed CP wave, respectively. The calculated co- and cross- polarized reflectances, $R_{ij} = |r_{ij}|^2, i, j = x, y$, and transmittances, $T_{ij} = |t_{ij}|^2$, for our structure are depicted in Fig. S1. We observe that four out of eight scattering amplitudes are zeros ($t_{-+}, t_{+-}, r_{++}, r_{--}$), as expected for bi-isotropic chiral structures. The first resonance, at 212 THz, is associated with dip in transmittance t_{--} and peak in t_{++}. This resonance is coming from the antiparallel currents (a typical magnetic resonance) on the top and bottom crosses (back and front crosses in Fig. 1) as shown by eigenmode simulations (see Fig. S4). The second resonance

(around 280 THz) is predominantly the electric dipole resonance of the parallel to the electric field wire elements of the crosses. The confirmation of electric and magnetic nature of the resonances is also verified by a retrieval procedure (see Section S2).

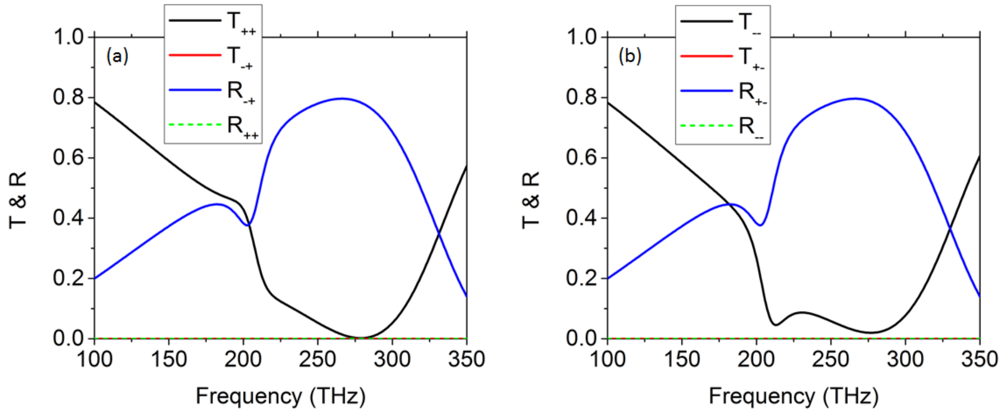

Figure S1: Transmittances (T) and reflectances (R) as a function of frequency for the chical metasurface of Fig. 1 in the main text, when illuminated (a) by LCP/- waves and (b) by RCP/+ waves.

To evaluate the chiral response of our structure, we calculate the transmitted wave ellipticity, $\eta = 0.5 \tan^{-1} [(|t_{++}|^2 - |t_{--}|^2)/(|t_{++}|^2 + |t_{--}|^2)]$, directly connected to the circular dichroism, $CD = |t_{++}|^2 - |t_{--}|^2$, as well as the polarization rotation angle, $\theta_{act} = (1/2)[\arg(t_{++}) - \arg(t_{--})]$, a measure of the optical activity. using the transmission data of Eq. (S2). The corresponding results are shown in Fig. S2; they reveal that in the absence of the gain the ellipticity is close to 20° while in the present of gain (pump on, $R_p = 2 \times 10^8$ s^{-1}) it reaches almost 45° which indicates pure CP waves. Furthermore, we observe a similar enhancement in the optical activity, from 15° to above 30°, demonstrating once again the strong coupling between the meta-atom and the gain.

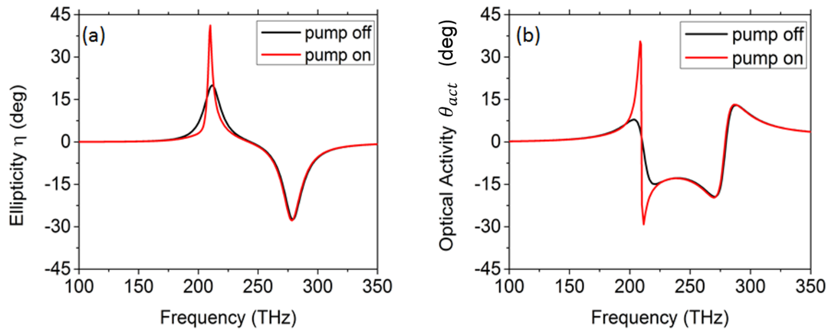

Figure S2: Transmitted wave ellipticity, η, and optical activity, θ_{act}, as a function of frequency for the structure of Fig. 1 in the main text. The black line corresponds to the passive chiral metasurface while the red line corresponds to the chiral metasurface with gain (pump rate $R_p = 2 \times 10^8 s^{-1}$)

Next, we discuss the sensitivity of the electromagnetic response of our structure on different geometrical parameters, and in particular the effect of changing the rotation angle between the crosses on the scattering features. The reflectances and transmittances for twist angle $\theta = +22.5°$ and $\theta = -22.5°$ between top/front and bottom/back cross, as well as the corresponding ellipticity, η, and optical activity, θ_{act}, are illustrated in Fig. S3. By tuning the relative twist angle from +22.5 to -22.5 an interchange between RCP and LCP transmitted waves is observed leaving unaffected the reflected wave.

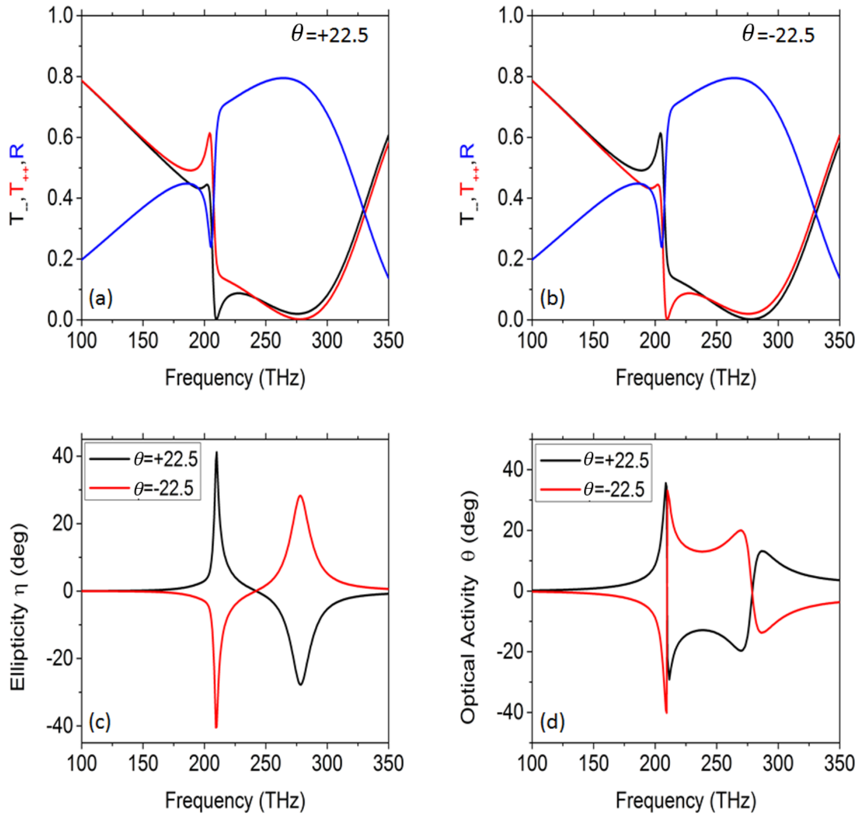

Figure S3: Scattering amplitudes (reflectances, R, and tranmittances, T) for the structure of Fig. 1 of the main text for two different relative twist angles of the crosses; (a) for $\theta = +22.5°$ and (b) for $\theta = -22.5°$. Panels (c) and (d) shows the corresponding elipticities, η, and optical activities, θ_{act}, respectively.

The response of our chiral metamaterial when illuminated by a linearly polarized plane wave can be understood by examining the profile of the elecromagnetic eigenmodes of the structure. Here we perform an eigenmode analysis in order to identify the eigenmodes in the vicinity of the fundamental (lasing) frequency. This way, we can unequivocally identify the metasurface modes that mediate the interaction of the incident and generated waves with the metasurface. The eigenmodes (if excited) are associated with well-defined scattering (transmission-reflection) resonances. We identified two degenerate eigenmodes close the fundamental (lasing) frequency, and specifically at 213.98 THz. The electric field component of the modes profile (eigenvector) is depicted in Fig. S4. In particular, we show the absolute

value of the electric field for the chiral metamaterial without gain, panels (a)-(b), and the z-component of the electric field, panels (c)-(d), at the middle-plane of the crosses (xy cross-section). From the field profile of Figs. S4 one can conclude the presence of antiparallel currents in the top and bottom layers, resulting to asymmetric (magnetic) resonance modes. Each of the two degenerate modes concerns excitation of one of the two "quasi-parallel" arms of the cross-pair. (The chirality of the structure comes by twisting the crosses, breaking the structure mirror-symmetry.)

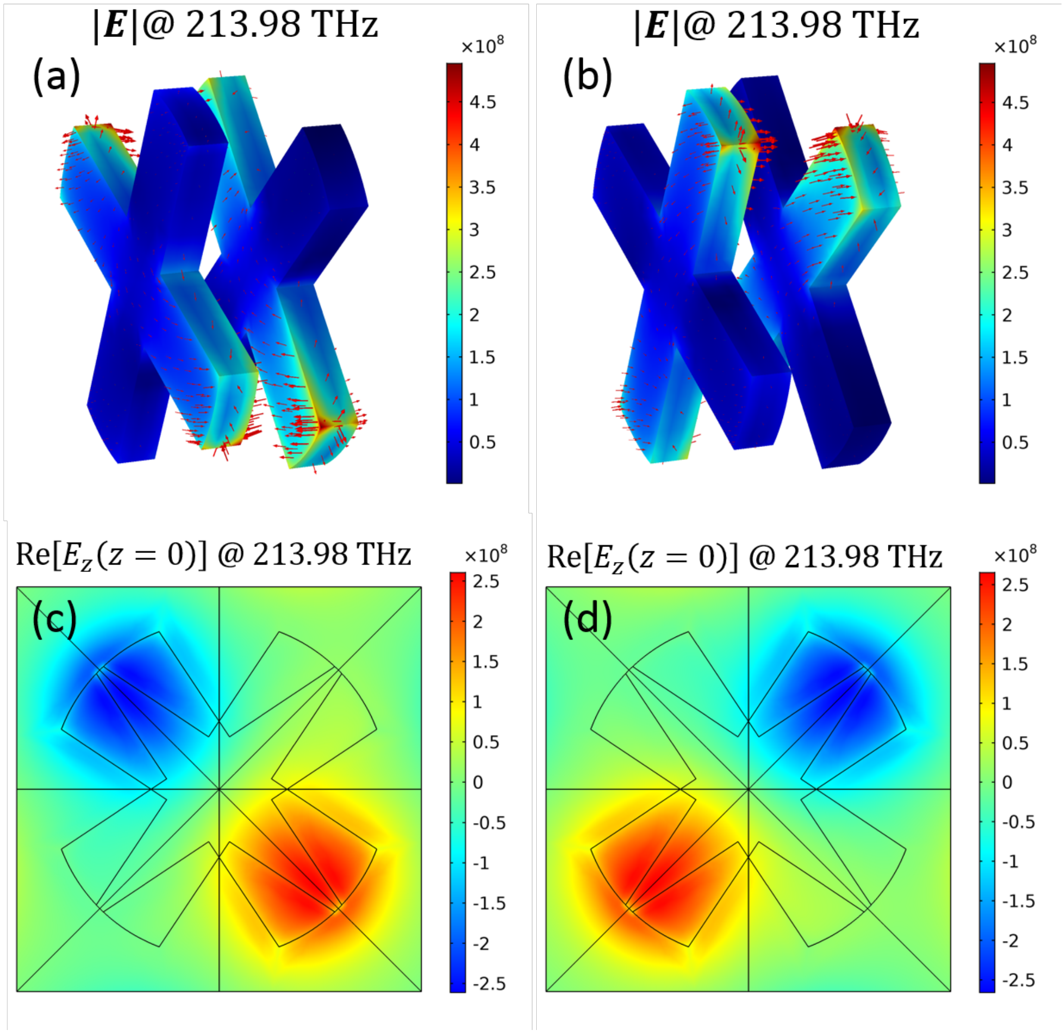

Figure S4: Electric field distributions (obtained from eigenmode analysis) for the two-degenerate eigenmodes of our structure at the fundamental (lasing) frequency. Panes (a) and (b) show the absolute value of the electric field (in V/m). Panels (c) and (d) show the z-component of the electric field at the middle-plane between the crosses (xy cross-section).

S2 Retrieval procedures

Given that our metasurface thickness is much smaller than the operating wavelength, it can be described as a thin sheet that extends on xy-plane and supports electric and magnetic currents. Taking into account that the metasurface is also isotropic, then the conductivity (σ) tensor related to the local surface current densities and local fields is expressed as

$$\begin{bmatrix} j_e^{(x)} \\ j_e^{(y)} \\ j_m^{(x)} \\ j_m^{(y)} \end{bmatrix} = \begin{bmatrix} \sigma_{ee} & 0 & \sigma_{em} & 0 \\ 0 & \sigma_{ee} & 0 & \sigma_{em} \\ \sigma_{me} & 0 & \sigma_{mm} & 0 \\ 0 & \sigma_{me} & 0 & \sigma_{mm} \end{bmatrix} \begin{bmatrix} E_{loc}^{(x)} \\ E_{loc}^{(y)} \\ H_{loc}^{(x)} \\ H_{loc}^{(y)} \end{bmatrix}, \tag{S3}$$

where $\mathbf{j_e}$ and $\mathbf{j_m}$ are the surface electric and surface magnetic current densities, respectively, and the subscript loc in the fields indicates local fields on the metasurface. Using Eq. (S3), we solve Maxwell's equations with the appropriate boundary conditions, which are $\mathbf{n} \times (\mathbf{E}_2 - \mathbf{E}_1) = -\mathbf{j}_m$, $\mathbf{n} \times (\mathbf{H}_2 - \mathbf{H}_1) = +\mathbf{j}_e$, with $\mathbf{n}$ the unit-vector normal of the sheet from region 1 (before the metasurface) to region 2 (i.e. pointing to $+z$). Assuming that $\sigma_{em} << \sigma_{ee}, \sigma_{mm}$, so eliminating terms $\sim \sigma_{em}^2$, and replacing the local fields by average field values, i.e. $E_{loc}^{(i)} = (E_1^{(i)} + E_2^{(i)})/2, i = x, y$, etc., we find the reflection and transmission amplitudes in terms of $\sigma_{ee}, \sigma_{mm}, \sigma_{em}$, as

$$t_{xx} = t_{yy} = \frac{\zeta - Z_2(\sigma_{ee}/2)(\sigma_{mm}/2Z_1)}{(\zeta+1)/2 + (Z_2\sigma_{ee}/2) + (\sigma_{mm}/2Z_1) + [(\zeta+1)/2\zeta](Z_2\sigma_{ee}/2)(\sigma_{mm}/2Z_1)} \tag{S4}$$

$$t_{xy} = -t_{yx} = \frac{\zeta\sigma_{em}}{(\zeta+1)/2 + (Z_2\sigma_{ee}/2) + (\sigma_{mm}/2Z_1) + [(\zeta+1)/2\zeta](Z_2\sigma_{ee}/2)(\sigma_{mm}/2Z_1)} \tag{S5}$$

$$r_{xx} = r_{yy} = \frac{(\zeta-1)/2 - (Z_2\sigma_{ee}/2) + (\sigma_{mm}/2Z_1) + [(\zeta-1)/2\zeta](Z_2\sigma_{ee}/2)(\sigma_{mm}/2Z_1)}{(\zeta+1)/2 + (Z_2\sigma_{ee}/2) + (\sigma_{mm}/2Z_1) + [(\zeta+1)/2\zeta](Z_2\sigma_{ee}/2)(\sigma_{mm}/2Z_1)} \tag{S6}$$

$$r_{xy} = r_{yx} = 0 \tag{S7}$$

where $\zeta = Z_2/Z_1$, with Z_1, Z_2 the wave impedance in regions 1 and 2, respectively, i.e. before and after the metasurface. Inverting the above equations we take the dimensionless conductivities in terms of reflection and transmission amplitudes, as

$$s_{ee} = \frac{Z_2\sigma_{ee}}{2} = \frac{\zeta - \zeta r_{xx} - t_{xx}}{1 + r_{xx} + t_{xx}} \tag{S8}$$

$$s_{mm} = \frac{\sigma_{mm}}{2Z_1} = \frac{\zeta + \zeta r_{xx} - \zeta t_{xx}}{\zeta - \zeta r_{xx} + t_{xx}} \tag{S9}$$

$$s_{em} = \frac{2t_{yx}(1 + \zeta + r_{xx} + \zeta r_{xx})}{(1 + r_{xx} + t_{xx})(\zeta - \zeta r_{xx} + t_{xx})}. \tag{S10}$$

Another, also important, approach to derive effective parameters for metamaterial slabs is based on bulk retrieval homogenization process [1], which derives bulk effective parameters, i.e. permittivity, permeability and chirality, from reflection and transmission data. We should stress here that homogenization, which suggests that effective optical parameters can be defined for composite media with subwavelength-sized meta-atoms, is a fundamental concept in metamaterials.

Assuming a slab (of sub-wavelength thickness d) of reciprocal (Pasteur) chiral response and applying the conditions of continuity of tangential electric and magnetic fields at the boundaries, $z = 0$ and $z = d$, we obtain the transmission and reflection coefficients [1] as

$$t_{\pm} = \frac{4Ze^{in\omega d/c}e^{\pm i\kappa\omega d/c}}{(1+Z)^2 - (1-Z)^2 e^{2in\omega d/c}}, \tag{S11}$$

$$r_{+-} = r_{-+} = r = \frac{(1-Z^2)(e^{2in\omega d/c} - 1)}{(1+Z)^2 - (1-Z)^2 e^{2in\omega d/c}}. \tag{S12}$$

where $t_+ \equiv t_{++}$, $t_- \equiv t_{--}$ and Z is the wave impedance in the "bulk" (slab) metamaterial. Inverting the above equations, the impedance Z and the refractive indices $n_{\pm}$ (for right-handed (+) and left-handed (-) CP waves) are expressed as

$$Z = \pm\sqrt{\frac{(1+r)^2 - t_+t_-}{(1-r)^2 - t_+t_-}}, \tag{S13}$$

$$n_{\pm} = \frac{ic}{\omega d}[\ln[\frac{1}{t_{\pm}}(1 + \frac{Z-1}{Z+1}r_{\pm})] \pm 2m\pi]. \tag{S14}$$

where m is an integer determined by the branches. Then the effective material parameters ϵ, μ and κ can be obtained with the following relations: $n_{av} = (n_+ + n_-)/2, \kappa = (n_+ - n_-)/2, \varepsilon = n_{av}/Z$ and $\mu = n_{av}Z$.

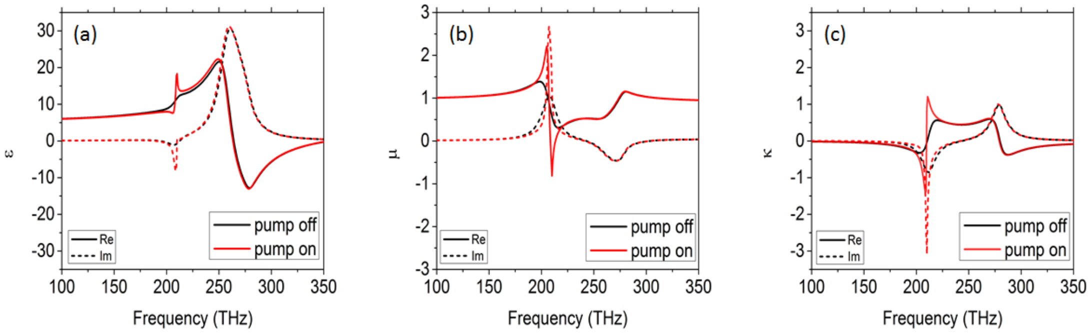

Figure S5: Real and imaginary parts of the effective material parameters of our structure. Panels (a), (b),(c) show the electric permittivity, ε, magnetic permeability, μ, and chirality parameter, κ, respectively. The black lines correspond to passive metasurface (without gain) while red lines correspond to metasurface with gain ($R_p = 2 \times 10^8 s^{-1}$).

The real and imaginary part of the effective chirality parameter is given as

$$\mathrm{Re}(\kappa) = \frac{\arg(t_+) - \arg(t_-) + 2m\pi}{2k_0 d}, \tag{S15}$$

$$\mathrm{Im}(\kappa) = \frac{\ln|t_+| - \ln|t_-|}{2k_0 d}, \tag{S16}$$

where $k_0 = \omega/c$.

Using the above expressions we extract the bulk effective material parameters of our structure with and without gain. Figure (S5) shows the retrieved results for the real and the imaginary parts of the effective electric permittivity, magnetic permeability and chirality parameter with gain (pump rate $R_p = 2 \times 10^8\ \mathrm{s}^{-1}$) and without gain. We observe that with the incorporation of gain the weak and broad response of the metasurface becomes strong and narrower, resulting to un-damping of the metasurface resonance, a behavior qualitatively similar to the one shown in the main text for the surface conductivities.

Lastly we calculate the effective parameters for two different twist angles between the crosses, $\phi = +22.5°$ and $\phi = +22.5°$. We find that the effective electric permittivity and magnetic permeability do not change by reversing the twist angle; the chirality parameter though changes sign in both real and imaginary parts, as shown in Fig. S6.

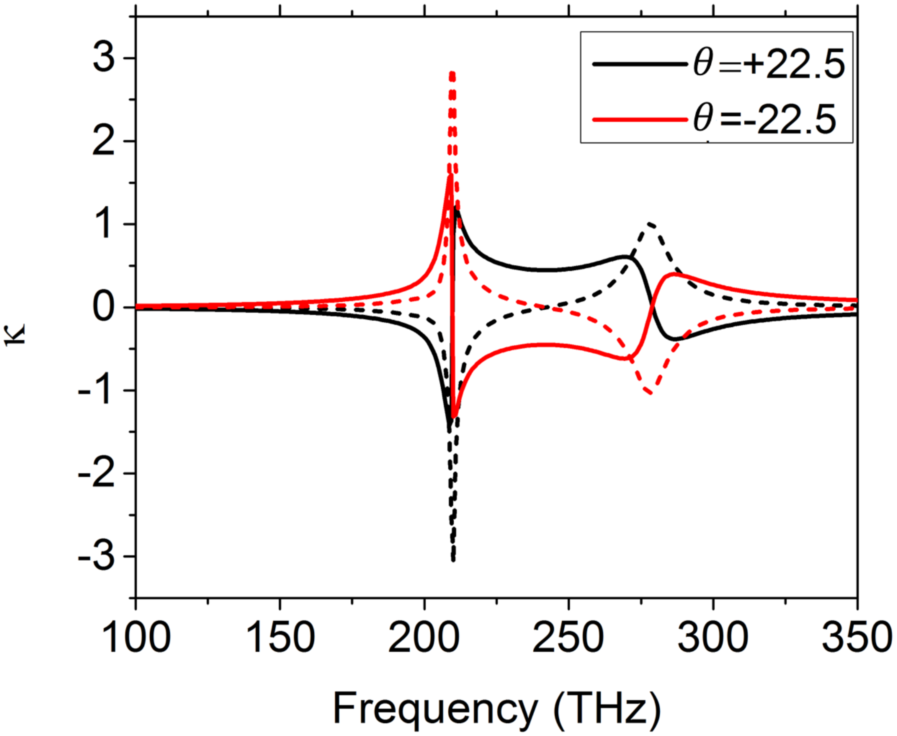

Figure S6: Real (solid lines) and imaginary (dashed lines) parts of the effective chirality parameter for the two opposite twist angles examined, for our metasurface with gain. Black lines are for $\theta = +22.5°$ while red lines for $\theta = -22.5°$.

S3 Additional results verifying the dynamic lasing polarization control possibility

To examine further the impact of the incident wave polarization angle on the output wave polarization, we calculated the RCP/+ and LCP/- transmitted waves for our chiral meta-atoms with continue wave (CW) excitation. The results are shown in Fig. S7. Observing the results of Figs. S7, we see that $\phi = 0$ deg the target lasing mode is dominated by LCP/-, with negligible RCP/+ radiation emission. Twisting the polarization angle by $\phi = 45$ deg things reverse and the main lasing mode is dominated now by RCP/+ radiation while LCP/- radiation emission is at the noise level. The intermediate angles are also shown in Figs. S7, where indicates the polarization controllable laser output.

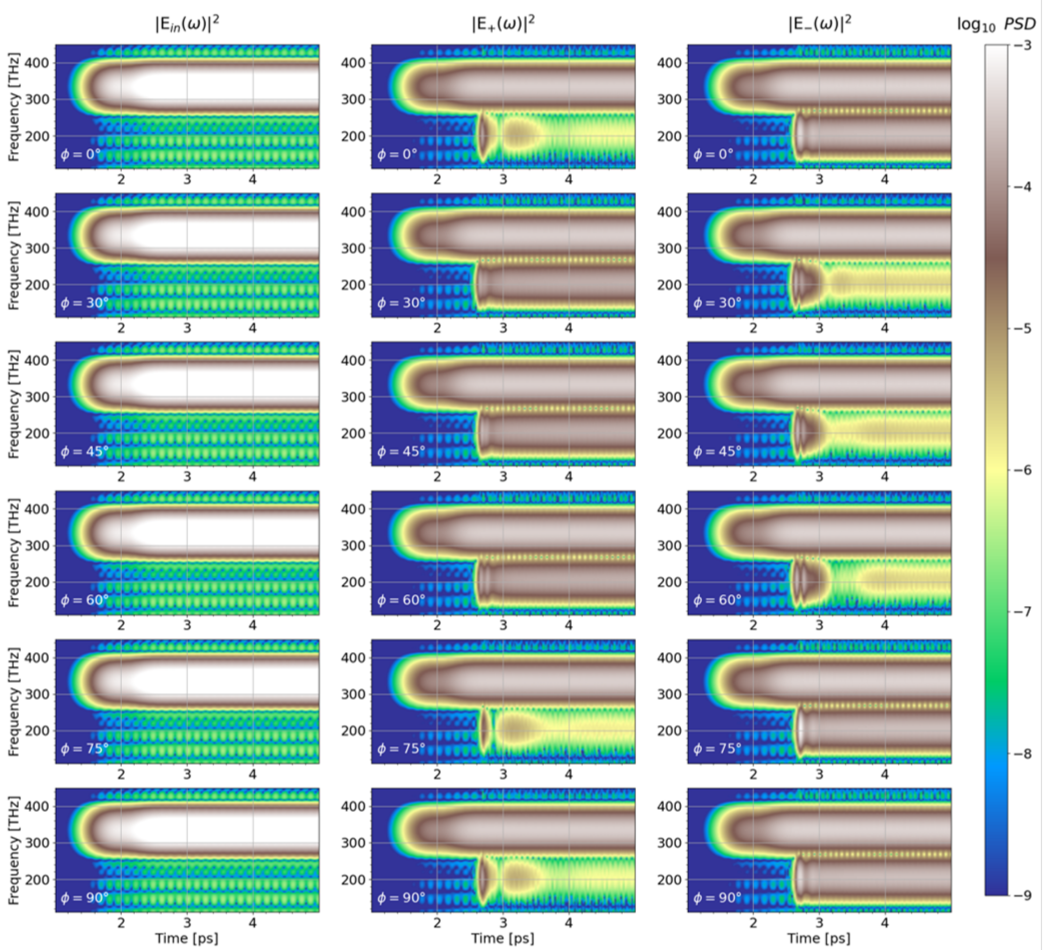

Figure S7: The incident and transmitted power spectral density from the structure shown in the main text for incidence of a continues linearly polarized wave (CW) of different polarization angles, ϕ) (in respect to the x-axis). First column corresponds to the incident, second column to the RCP transmitted and third column to the LCP transmitted, respectively, as a function of time and frequency. Each row corresponds to the different polarization angles, $\phi = 0$ deg , $\phi = 30$ deg, $\phi = 45$ deg, $\phi = 60$ deg , $\phi = 75$ deg and $\phi = 90$ deg, as shown in the legents.

In the above analysis and in the main text we show the demonstration of CP laser under CW excitation. Here, we pump the gain molecules with a short intensive Gaussian pump pulse with a central frequency of $\omega_b = 2\pi \times 335 \times 10^{12}$ rad/s and duration $t_p = 2 \times \texttt{FWHM} =$ 2ps, where $\texttt{FWHM}$ denotes the full-width at half maximum of the pulse; $\mathbf{E}_p(t) = (\hat{\mathbf{x}} \cos\phi + \hat{\mathbf{y}} \sin\phi) E_p \sin(\omega_b \tau_p) \exp\left[-\frac{1}{2}(\tau_p/\sigma_p)^2\right]$, where $\tau_p = t - t_p$ and $\sigma_p = \texttt{FWHM}/(2\sqrt{2\log 2})$. Figures S8(a)-(c) depict the incident, co-polarized transmitted and cross-polarized transmitted waves as a function of time for polarization angle $\phi = 45°$. Then, we Fourier transform the time-dependent transmitted electric fields to see if there is emission and how strong is the emitted radiation around $\omega_a = 2\pi \times 205 \times 10^{12}$ rad/s (lasing frequency). The results are depicted in Figs. 3(d)-(f). The pump-pulse amplitude, E_p, is 0.8×10^6 V/m and we do observe a peak at the emission frequency.

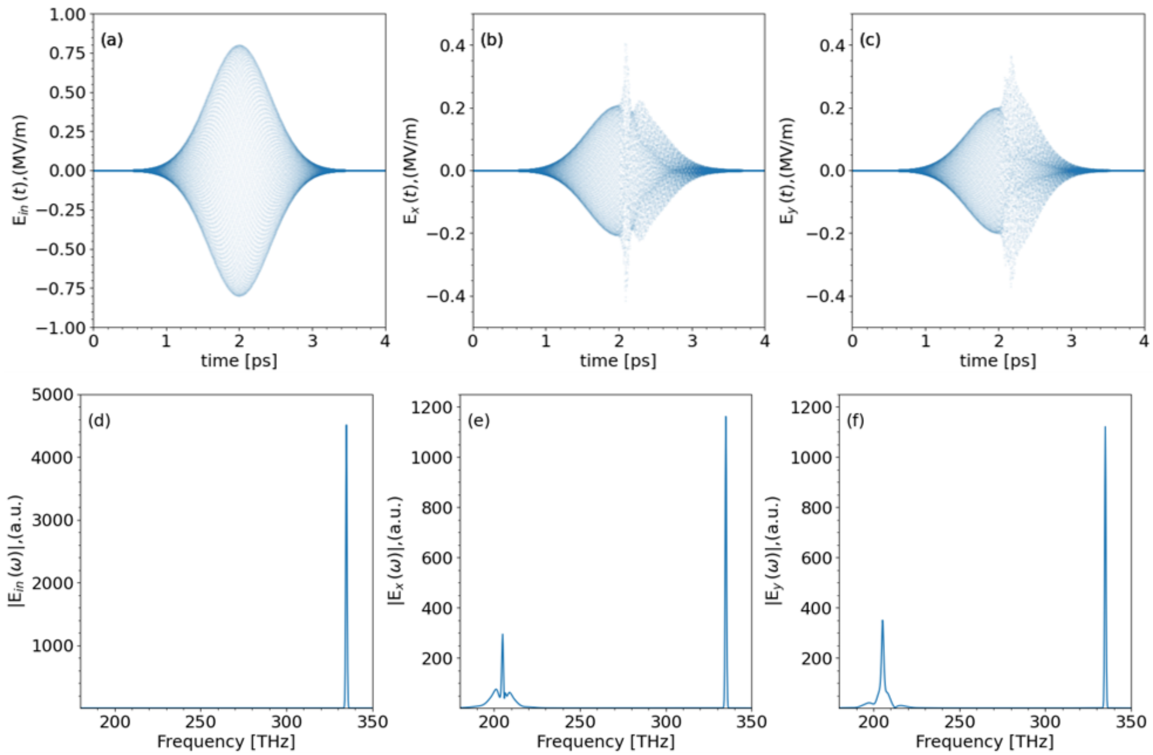

Figure S8: The incident and transmitted waves as a function of time for the structure of Fig. 1 in the main text excited by a pump pulse of frequency 335 THz and polarization angle $\phi = 45°$. Panels (a), (b) and (c) are the incident, transmitted E_x, and transmitted E_y waves for input pump-pulse amplitude 0.8×10^6 V/m. Panels (d), (e) and (f) are the corresponding Fourier transformed spectra of the incident and transmitted waves.

To investigate further the lasing wave polarization and examine the possibility of its control, we keep the same pump-pulse amplitude, 0.8×10^6 V/m, we modify the relative polarization angle, ϕ, from $\phi = 0°$, corresponding to linearly polarized incident pulse along x-axis, to $\phi = 90°$, corresponding to linearly polarized incident pulse along y-axis. The results for $\phi = 0°$ and $\phi = 45°$ are shown in Fig. S9, where the two rows denote the two different angle cases, and both input and output waves are transformed in the circular polarization basis. Figs. S9 (a), (d) show the power spectral density (in log scale) of the

incident, (b), (e) of the RCP transmitted (E_+) and (c), (f) of the LCP transmitted (E_-) waves. When the polarization angle is $\phi = 0°$ we observe that above 2.5 ps the E_+ polarized emitted wave vanishes, while the E_- polarized wave is the dominant, indicating the pure circularly polarized output. As we tune the polarization angle to $\phi = 45°$ (the incident pulse is now polarized parallel to the diagonal of the metasurface unit-cell), we observe an interchange between the E_+ and E_- transmitted waves at the lasing frequency; thus the dominant transmitted wave is the RCP, E_+, wave.

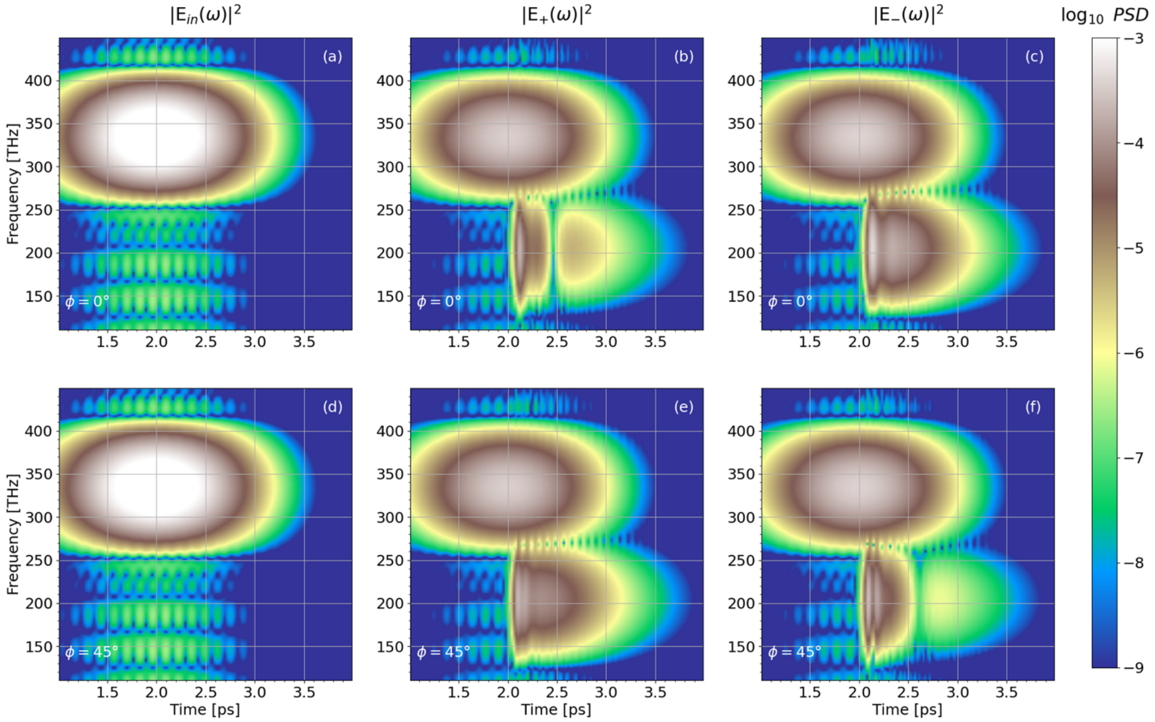

Figure S9: The incident and transmitted power spectral density (color) for different polarization angles. Panels (a), (b) and (c) show the incident, RCP transmitted, $|E_+|^2$, and LCP transmitted $|E_-|^2$ wave, respectively, as a function of time and frequency for incident polarization angle $\phi = 0°$. Panels (d), (e), (f) show the corresponding results for polarization angle $\phi = 45°$.

To examine further the nature of the lasing mode of our structure and in particular the change of the lasing from LCP to RCP as we change the pump/incident wave linear polarization from 0 to 45° we performed a detailed field analysis. Results of this analysis are shown in Figs. S10 and S11, where we illustrate the spatial distribution of the the z component of the electric field (lasing mode) at incident polarization angles $\phi = 0^o$ and $\phi = 45^o$, as in the Figure 5 in the main text. In Figure S10 we plot the spatial distribution of E_z for $\phi = 0^o$ at different time steps over one period. The results clearly demonstrate a counter-clockwise lasing mode. The opposite occurs in Fig. S11, where we plot E_z for $\phi = 45^o$, demonstrating a clockwise mode.

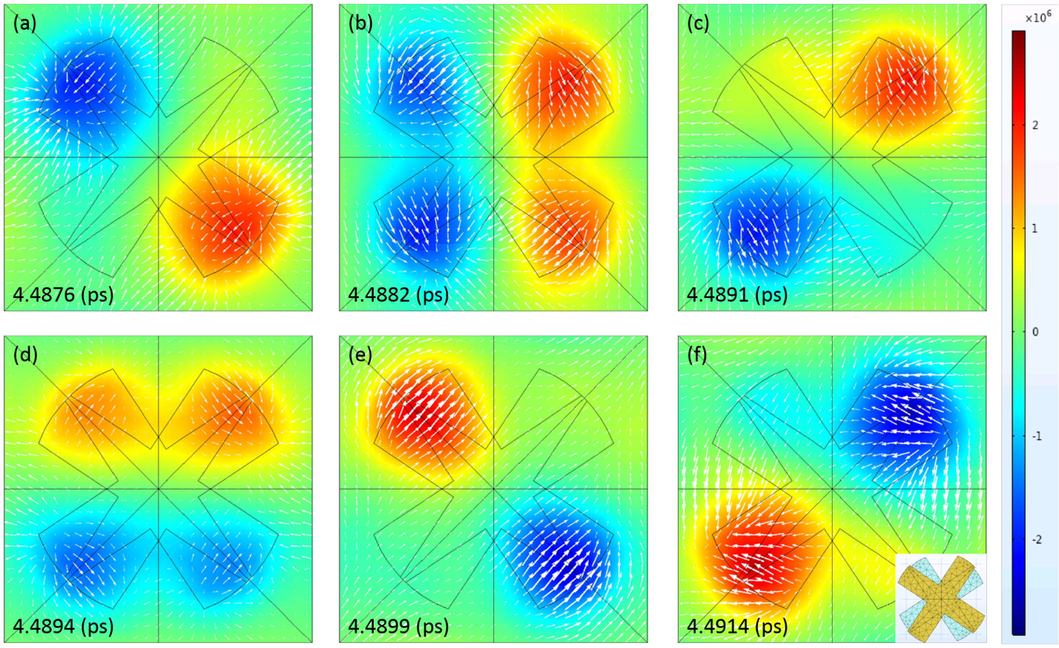

Figure S10: Panels (a)-(f) correspond to the electric field component E_z of the lasing mode for the unit cell shown in the inset, for incident polarization angle $\phi = 0^o$, in a parallel to the metallic crosses cross-section of the unit cell in the middle between the two crosses. The field is plotted at different time steps within one period (see different panels; time is indicated with the legends; it increases from left to right).

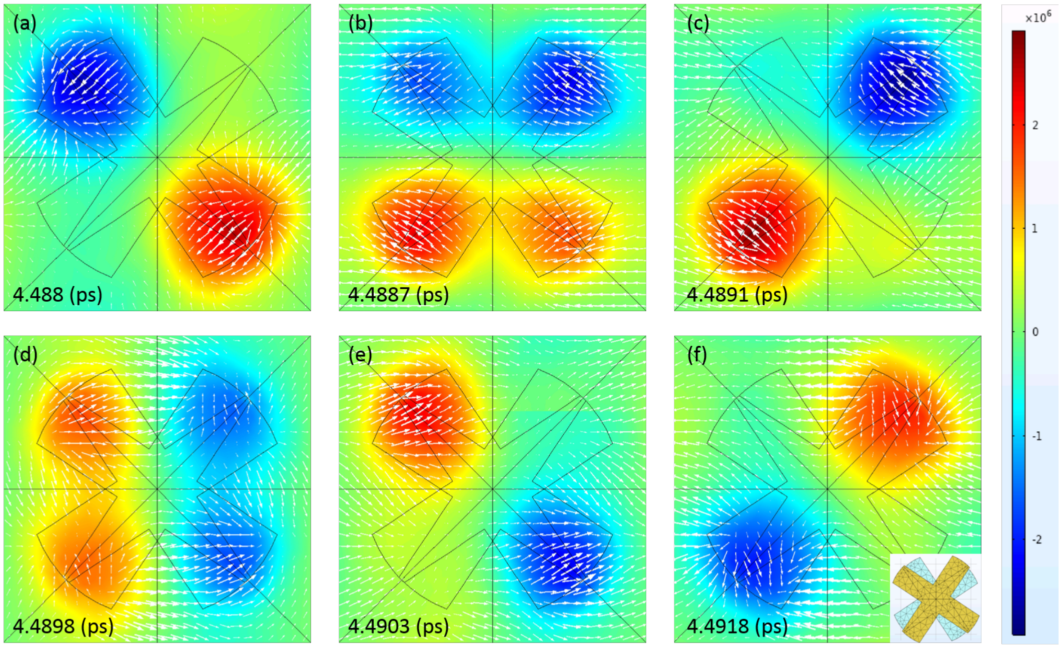

Figure S11: Panels (a)-(f) correspond to the electric field component E_z of the lasing mode for incident polarization angle $\phi = 45^o$ in the cross-section of the metasurface unit-cell at different time steps within one period. Details are as in Fig. S6.

S4 Derivation of macroscopic polarization through microscopic polarization of molecules

Here we outline the derivation of the dynamic response of the macroscopic polarization density describing the interaction of a gain medium (like dye molecules) with light, based on a basic atomic model. Gain molecules can be modeled as four-level systems which coherently absorb energy through optical pumping and emit energy at shorter frequencies. These processes (absorption and emission) require two optical transitions, one for the absorption (pump) and one for the emission (lasing). Assuming dipole-forbidden optical transition between the two processes, we can approach our four-level system as two subsystems of two-level atoms.

Therefore, in order to describe both absorption and emission we assume a collection of two-level systems (levels $|1\rangle$, ground, and $|2\rangle$, excited) coupled resonantly to the external electric field in the dipole approximation. The Hamiltonian which describes the system has the following form [2]:

$$\hat{H} = \hat{H}_0 + \hat{H}_{int} \equiv \hbar\omega_1|1\rangle\langle 1| + \hbar\omega_2|2\rangle\langle 2| - \mu_{12}\cdot\mathbf{E}(t)|1\rangle\langle 2| - \mu_{21}\cdot\mathbf{E}(t)|2\rangle\langle 1| \tag{S17}$$

where $\hbar\omega_1$ and $\hbar\omega_2$ are the energies of the ground and excited state, respectively, and μ_{12}, μ_{21} are the dipole moment elements of the electronic transition between the two states $|1\rangle$, $|2\rangle$. (Note that there is a state transition and transition dipole moment vector component for each spatial direction. In our analysis the two-level systems are spherically symmetric, without permanent dipole moments, hence the diagonal terms μ_{11}, μ_{22} are zero. Moreover we consider real dipole moments and electric fields.)

A wave-function for the above two-level system will be a superposition of the two states $|1\rangle$ and $|2\rangle$ (as concluded by time dependent perturbation theory), of the form

$$|\psi\rangle = c_1(t)|1\rangle + c_2(t)|2\rangle \tag{S18}$$

with $|c_i|^2, i = 1, 2$, the probability the system to be in state $|i\rangle$. The total electric dipole moment, $\mathbf{p}$, for the above system will be the average of the electric dipole operator $\hat{\mu} = -e\mathbf{r}$ with the state $|\psi\rangle$, which can be written as

$$\mathbf{p} = \langle\psi|\hat{\mu}|\psi\rangle = c_1^* c_2\langle 1|\hat{\mu}|2\rangle + c_1 c_2^*\langle 2|\hat{\mu}|1\rangle \equiv -e\mathbf{r}_{12}\rho_{21} - e\mathbf{r}_{21}\rho_{12} = -2e\mathbf{r}_{12}\mathrm{Re}(\rho_{12}) \tag{S19}$$

where $\mathbf{r}_{12} = \mathbf{r}_{21}$ are the electron position operator matrix elements and $\rho_{12} = \rho_{21}^*$ are the off-diagonal elements of the density matrix, which are called atomic coherences. From Eq. (S19) one concludes that the atomic polarization is the product of the dipole moment matrix elements with the atomic coherences. Hence, in order to calculate it (and through it the macroscopic polarization density) one needs to calculate the density operator matrix elements.

The density operator can represent a collection of identical systems in possibly different states, giving the probability the system to be in each state. For the current system it takes the form

$$\rho = |\psi\rangle\langle\psi| = \sum_{i,j} c_i c_j^* |i\rangle\langle j| = \sum_{i,j} \rho_{ij}|i\rangle\langle j|, \quad i, j = 1, 2, \tag{S20}$$

where $\rho_{ij} = c_i c_j^*$.

Differentiating the density operator and employing the Schrodinger equation, we can write the so-called von Neumann equation of the dynamics of the density operator:

$$i\hbar\dot{\rho} = [\hat{H}, \rho] = \hat{H}\rho - \rho\hat{H} \tag{S21}$$

From von Neumann equation, taking into account that $\rho_{12}^*(t) = \rho_{21}(t)$ and $\mu_{12} = \mu_{21} = \mu$ we obtain after simple algebra the following equations for the density-matrix elements:

$$\begin{aligned} \dot{\rho}_{12}(t) = \dot{\rho}_{21}^*(t) = i\Omega[\rho_{22}(t) - \rho_{11}(t)] + i\omega_{21}\rho_{12}(t), \\ \dot{\rho}_{22}(t) = -\dot{\rho}_{11}(t) = i\Omega[\rho_{12}(t) - \rho_{12}^*(t)], \end{aligned} \tag{S22}$$

where $\omega_{21} = \omega_2 - \omega_1$ and $\Omega = \boldsymbol{\mu} \cdot \mathbf{E}(t)/\hbar$ is connected to the Rabi frequency $|\boldsymbol{\mu} \cdot \mathbf{E}_0|/\hbar$ ($\mathbf{E}_0$ is the field amplitude). So far we have assumed an isolated system; however, in general the system has interaction with the environment, hence dissipative effects should be also taken into account. The usual method of achieving this in simple systems is to include in Equations (S21) phenomenological terms involving characteristic decay rates. In particular, we can write [2]

$$\begin{aligned} \dot{\rho}_{12}(t) = \dot{\rho}_{21}^*(t) = i\Omega[\rho_{22}(t) - \rho_{11}(t)] + [i\omega_{21} - \frac{\gamma}{2}]\rho_{12}(t), \\ \dot{\rho}_{22}(t) = -\dot{\rho}_{11}(t) = i\Omega[\rho_{12}(t) - \rho_{12}^*(t)] - \gamma\rho_{22}(t). \end{aligned} \tag{S23}$$

where γ is the decay rate. The above equations are called optical-Bloch equations. The real part of $\rho_{12}(t)$ represents the polarization, so by separating $\rho_{12}(t)$, Eq. (S23), into the real and imaginary part we obtain

$$\frac{d\mathrm{Re}[\rho_{12}(t)]}{dt} = -\frac{\gamma}{2}\mathrm{Re}[\rho_{12}(t)] - \omega_{21}\mathrm{Im}[\rho_{12}(t)] \tag{S24}$$

$$\frac{d\mathrm{Im}[\rho_{12}(t)]}{dt} = -\frac{\gamma}{2}\mathrm{Im}[\rho_{12}(t)] + \omega_{21}\mathrm{Re}[\rho_{12}(t)] + \Omega[\rho_{22}(t) - \rho_{11}(t)] \tag{S25}$$

By taking the time derivative of equation (S24), substituting the $d\mathrm{Im}[\rho_{12}(t)]/dt$ from equation (S25) and after some straightforward mathematical manipulations we obtain

$$\frac{d^2\mathrm{Re}[\rho_{12}(t)]}{dt^2} = -\frac{\gamma}{2}\frac{d\mathrm{Re}[\rho_{12}(t)]}{dt} - \omega_{21}\{-\frac{\gamma}{2}\mathrm{Im}[\rho_{12}(t)] + \omega_{21}\mathrm{Re}[\rho_{12}(t)] + \Omega[\rho_{22}(t) - \rho_{11}(t)]\} \tag{S26}$$

Then we eliminate $\mathrm{Im}[\rho_{12}(t)]$ from equation (S24) and we take a second order differential equation in the form

$$\frac{d^2\mathrm{Re}[\rho_{12}(t)]}{dt^2} + \gamma\frac{d\mathrm{Re}[\rho_{12}(t)]}{dt} + (\omega_{21}^2 + \frac{\gamma^2}{4})\mathrm{Re}[\rho_{12}(t)] = -\omega_{21}\Omega[\rho_{22}(t) - \rho_{11}(t)] \tag{S27}$$

Taking into account that the electric dipole moment $\mathbf{p}$ is expressed as $\mathbf{p} = 2\boldsymbol{\mu}\mathrm{Re}(\rho_{12})$ (S19) and substituting the frequency $\Omega = \boldsymbol{\mu} \cdot \mathbf{E}(t)/\hbar$ we obtain

$$\frac{d^2\mathbf{p}}{dt^2} + \gamma\frac{d\mathbf{p}}{dt} + (\omega_{21}^2 + \frac{\gamma^2}{4})\mathbf{p} = -\frac{2\omega_{21}\mu^2}{\hbar}\mathbf{E}(t)[\rho_{22}(t) - \rho_{11}(t)] \tag{S28}$$

which is formally analog to the real macroscopic polarization equations [(5) in the main text] with $\sigma = \frac{2\omega_{21}\mu^2}{\hbar}$ the coupling strength in units $[C^2/kg]$.

S5 Simulation method

For a full-wave solution of the Maxwell's equations for the composite active chiral metamaterial we use finite-element time domain (FETD) simulations (via the COMSOL Multiphysics software). From the simulations we derive the transmitted electric field when a wave is normally incident on a meta-atom incorporating active (quantum) gain molecules.

A visual representation, in the form of a flow chart, of the calculations involved in the time dependent simulations is shown in Fig. S12. Maxwell's equations are solved first, after which the electric field is supplied in rate equations, which are solved and the resulting population differences are then used to calculate the polarizations. The polarizations are fed back into Maxwell's equations and the process is repeated. COMSOL Multiphysics finite element simulations utilize a free tetrahedral mesh with distance between data points ranging from 12 nm to 90 nm and time discretization $dt = 9 \times 10^{-17}$ s. The maximum element growth rate is chosen as 1.5, the curvature factor is chosen as 0.6, and the relative tolerance as 0.001. Note that anticipated variations in the system, including those associated with physical noise or small computational errors, are expected.

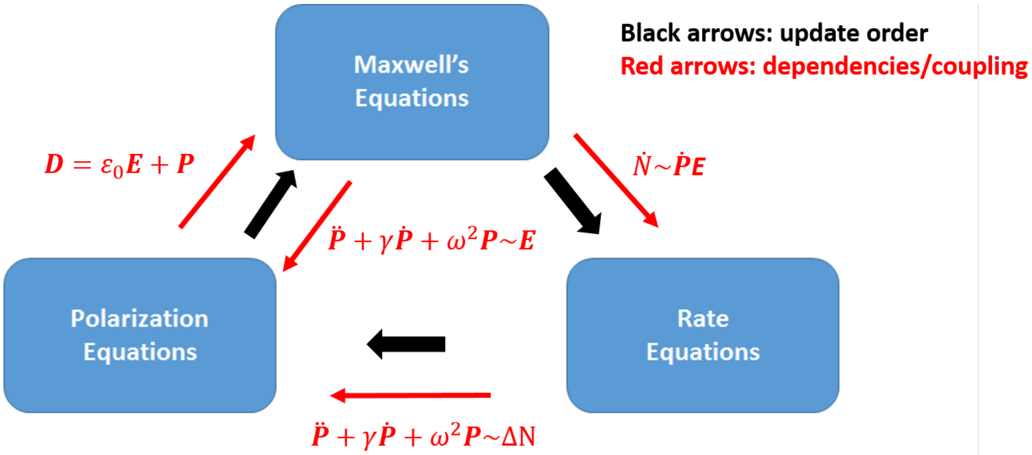

Figure S12: A flow chart showing the calculation taking place in the time-dependent simulations of gain-bearing metamaterials. Maxwell's equations are solved first, after which the electric field is supplied to the rate equations that are then used to feed with the population-differences the polarization equations and calculate the polarization. The polarization is fed back into Maxwell's equations and the process is repeated.

References

[1] R. Zhao, T. Koschny, C. M. Soukoulis, *Chiral metamaterials: Retrieval of the effective parameters with and without substrate*, Optics Express, **84**, 14553 – 14567, (2010).

[2] Lambropoulos, P.; Petrosyan, D. *Fundamentals of Quantum Optics and Quantum Information*, Springer Berlin Heidelberg: Berlin, Heidelberg, 2007.
